# Measurement of the Population of Electrosprayed Deprotomers of Coumaric Acids Using UV–Vis Laser Photodissociation Spectroscopy

**DOI:** 10.1021/acs.jpca.1c04880

**Published:** 2021-08-03

**Authors:** Natalie G. K. Wong, Conor D. Rankine, Caroline E. H. Dessent

**Affiliations:** † Department of Chemistry, University of York, Heslington, York YO10 5DD, U.K.; ‡ School of Natural and Environmental Sciences, Newcastle University, Newcastle-upon-Tyne NE1 7RU, U.K.

## Abstract

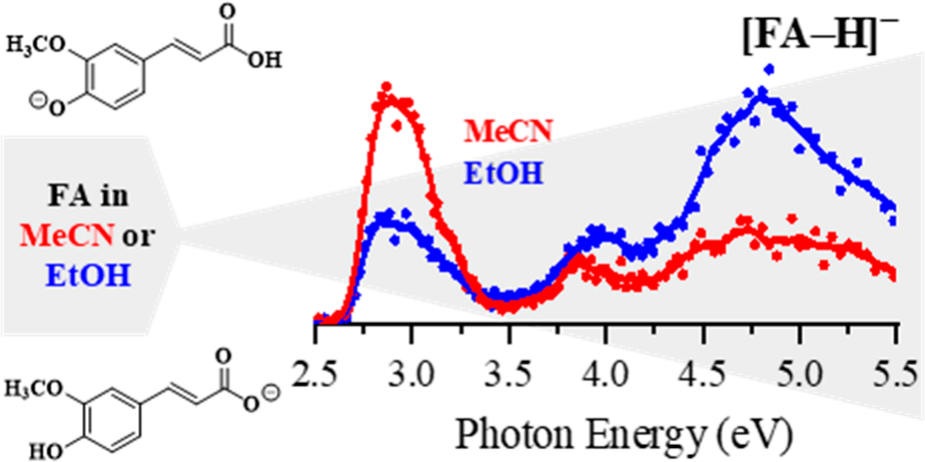

The measurement of deprotonation sites in multifunctional molecules following electrospray ionization is important to better inform a wide range of spectroscopic and photophysical studies that use electrospray to prepare molecular species for study in the gas phase. We demonstrate that low-resolution UV–vis laser photodissociation spectroscopy can be applied *in situ* to identify the deprotomers of three coumaric acids, *trans-para*-coumaric acid (CMA), *trans-*caffeic acid (CA), and *trans-*ferulic acid (FA), formed via electrospray. Electronic absorption spectra of the deprotonated coumaric acids are recorded via photodepletion and photofragmentation following electrospray from solutions of ethanol and acetonitrile. By comparing the experimental spectra to wave function theory calculations, we are able to confirm the presence of phenoxide and carboxylate deprotomers upon electrospray for all three coumaric acids, when sprayed from both protic and aprotic solvents. Ratios of the phenoxide:carboxylate deprotomers are obtained by generating summed theoretical absorption spectra that reproduce the experimental spectra. We find that choice of electrospray solvent has little effect on the ratio of deprotomers obtained for deprotonated CMA and CA but has a greater impact for FA. Our results are in excellent agreement with previous work conducted on deprotonated CMA using IR spectroscopy and demonstrate that UV photodissociation spectroscopy of electrosprayed ions has potential as a diagnostic tool for identifying deprotomeric species.

## 1 Introduction

The influence of electrospray ionization (ESI) conditions on the location of protonation and deprotonation sites of electrosprayed ions is a topic of keen debate.^1−13^ Acid–base reactions are of key importance throughout chemistry and biology, so correctly identifying the structures of protomers and deprotomers can be crucial to understanding reactive processes. While ESI has been successfully employed across analytical chemistry for many years, it is increasingly being used to probe solution-phase reactions and reactive intermediates for both chemical and biochemical systems.^14−17^ The role of the electrospray process in determining the location of protonation and deprotonation sites is therefore of key chemical interest.

Roithova and co-workers performed what is perhaps the seminal investigation of how the electrospray process affects the gas-phase ratios of deprotonated isomers by studying the *para*-hydroxybenzoic acid molecule.^13^ NMR was used to probe the solution-phase structures and ion-mobility mass spectrometry (IM-MS) was used to identify the gas-phase isomers. They were able to show that while the carboxylate isomer is preferred in solution irrespective of the solvent, the opposite is true for the gas-phase isomers. However, the exact ratio of isomers formed in the gas phase was found to depend strongly on the ESI solvent, pH, and solution concentration.^5,7,11,13^ These results led Roithova and co-workers to conclude that the gas-phase populations do not accurately reflect solution-phase populations, a conclusion that has been confirmed by a number of subsequent studies. As a consequence, it is very important to have tools available to determine accurately the identity of protonation/deprotonation isomers and also to have a full understanding of how the gaseous population relates to the solution-phase population as a function of the experimental conditions. Toward this end, recent studies have investigated the possibility of applying *in situ* spectroscopy to identify correctly electrosprayed protomers or deprotomers.^2,4,6−8,11^

**Scheme 1:**
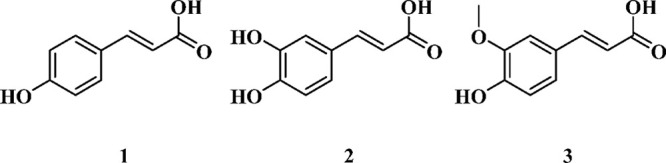
Schematic Diagram of **(1)***trans-para*-Coumaric Acid (CMA), **(2)***trans-*Caffeic Acid (CA), and **(3)***trans-*Ferulic Acid (FA)

In this work, we present a combined UV–vis laser photodissociation spectroscopy and quantum chemical study of a series of electrosprayed deprotonated coumaric acids (Scheme 1): namely, *trans-para*-coumaric acid (CMA), *trans-*caffeic acid (CA), and *trans-*ferulic acid (FA). The *para*-hydroxycinnamate unit is widespread in nature, being found as the chromophore in photoactive yellow protein^18^ and as sunscreen molecules in plants.^19^ Given their biological importance, deprotonated coumaric acids have been the focus of a number of recent gas-phase studies investigating their intrinsic photophysics and photochemistry.^20−22^ Here, our goal is to identify which deprotomers are present as a function of electrospray solvent through applying a combination of theory and experiment. We also aim to assess the potential of low-resolution UV–vis photodissociation as an analytical tool for identifying gaseous deprotomers. UV laser photodissociation spectroscopy should be highly sensitive to the electronic chromophore of the coumaric acid moiety, thus allowing us to identify unambiguously which isomers were present in those previous studies, and for electrosprayed coumaric acids generally.

CMA, CA, and FA are widely used as antioxidant agents in the cosmetic industry, leading to their long-standing use in antiaging cosmetics. They also absorb strongly in the UVA (400–320 nm) and UVB (320–280 nm), making them viable sunscreen agents.^23^ Antioxidants have considerable synergistic potential as components of sunscreen formulations since, in addition to their UV filtering ability, they can also function to protect against photoinduced radical reactions stemming from organic sunscreen molecules.^24−30^ Indeed, FA is already approved as a UV filter in Japan.^31^

The potential utility of coumaric acids as sunscreens has led to a number of studies of the fundamental photophysics of CMA to explore its excited state nonradiative relaxation pathways.^20−22,27,28,32,33^ Laser spectroscopic studies on the deprotonated forms of the CA and FA antioxidants are sparse, with only solution-phase transient absorption studies having been conducted to date.^27^ This is despite the fact that sunscreens are exposed to varying pH environments in common usage, with seawater and salt water lakes being alkaline, for example.^34,35^ Recent photochemical studies on how pH environment could affect the sunscreen performance at the molecular level have demonstrated that the deprotonated forms of oxybenzone, 2-phenylbenzimidazole-5-sulfonic acid, and benzophenone-4 are all able to photogenerate free radicals species via active photodecay channels in the gas phase.^36−38^ It is therefore important to investigate the deprotonated forms of the antioxidants studied here in the same context.

## 2 Methods

Gas-phase UV–vis photodissociation experiments were conducted in an AmaZon SL dual funnel ESI quadrupole ion-trap mass spectrometer (Bruker Daltonics Inc., Billerica, MA, USA), which was modified to allow laser-interfaced mass spectrometry (LIMS). This instrument has the advantages of a commercial mass spectrometer, coupled with the ability to record UV–vis photodissociation spectra in a routine manner.^39^

*trans-para*-Coumaric acid (4-hydroxycinnamic acid) and *trans*-caffeic acid ((2*E*)-3-(3,4-dihydroxyphenyl)acrylic acid) were purchased from Fluorochem Ltd. (Hadfield, Derbyshire, UK). *trans*-Ferulic acid ((2*E*)-3-(4-hydroxy-3-methoxyphenyl)prop-2-enoic acid) was purchased from Sigma-Aldrich, Inc. (St. Louis, MO, USA). HPLC-grade EtOH and MeCN were purchased from Fisher Scientific, Inc. (Pittsburgh, PA, USA), all used as received. Solutions of CMA, CA, and FA (1 × 10^–5^ M) in EtOH or MeCN were introduced into the mass spectrometer by ESI using typical instrumental parameters: nebulizing gas pressure 14.0 psi; injection rate 0.33 mL h^–1^; drying gas flow rate 10.0 L min^–1^; and run in the negative ion mode at a capillary temperature of 160 °C to form deprotonated ions. Trace amounts of NH_3_ solution (0.4%) was added to aid deprotonation.

[CMA-H]^−^, [CA-H]^−^, and [FA-H]^−^ were mass selected at *m*/*z* 163, 179, and 193, respectively, and isolated in the ion trap prior to laser irradiation. UV–vis photons were produced by a 10 Hz Nd:YAG (Surelite, Amplitude Laser Group, San Jose, CA, USA) pumped OPO (Horizon, Amplitude Laser Group) laser, giving ∼0.3 mJ across the range 496–224 nm (2.5–5.5 eV). Laser step sizes of 4 and 2 nm were used for that of excitations in the visible and UV regions, respectively. The laser beam was focused as has been described previously.^37,39^

Photofragmentation (PF) experiments were conducted with an ion accumulation time of 10 ms and a fragmentation time of 100 ms, thereby ensuring that each mass-selected ion packet interacted with one laser pulse to minimize the likelihood of multiphoton events. Multiphoton events via instantaneous absorption of multiple photons in the Franck–Condon region are negligible as the laser beam is only softly focused through the ion-trap region. In the limit where fluorescence is negligible,^40,41^ the UV-excited gaseous ion will fragment upon excited state relaxation, yielding an action absorption spectrum by photodepletion. Photodepletion (PD) of [CMA-H]^−^, [CA-H]^−^, and [FA-H]^−^ were measured as a function of the scanned wavelength, with the photofragment (PF) production also recorded simultaneously at each wavelength (see eqs 1a–1c):
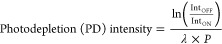
1a
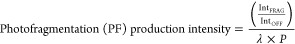
1b
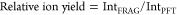
1cwhere Int_OFF_ and Int_ON_ are the peak parent ion intensities with laser off and on, respectively, Int_FRAG_ is the fragment intensity with the laser on, λ is the excitation wavelength (nm), *P* is the laser pulse energy (mJ), and Int_PFT_ is the sum of the photofragment ion intensities with the laser on. Photodepletion laser power dependence measurements are available in Section S3. PD intensities were taken from an average of three runs at each scanned wavelength. Fragment ions with *m*/*z* < 50 are not detectable in our mass spectrometer since low masses fall outside the mass window of the ion trap.

Electron detachment yield (ED*) spectra were calculated by assuming that any depleted ions not detected as ionic photofragments are decaying via means of electron detachment, as determined using eq 2a.^42^ This analysis assumes that both the parent ions and photofragments are detected equally in the mass spectrometer. In the figures where we present ED* spectra (Figures 5 and 6), we overlay such data with the photodepletion yield (PD*). PD* is the normalized photodepletion ion count (eq 2b), which provides the most straightforward comparison to the electron detachment yield (eq 2a).
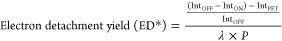
2a
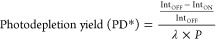
2bHigher-energy collisional dissociation (HCD) was employed to determine the ground state thermal fragmentation characteristics of [CMA-H]^−^, [CA-H]^−^, and [FA-H]^−^ in EtOH and in MeCN, using an Orbitrap Fusion Tribid mass spectrometer (Thermo Fisher Scientific, Waltham, MA, USA) with an ESI source, run in negative ion mode between 0 and 40% HCD energy, as described previously.^43,44^ This technique provides tandem mass spectrometer and was operated at a flow rate of 3.0 μL/min, with the following parameters: spray voltage, −3000 V; sheath gas flow rate, 3; auxiliary gas flow rate, 1; ion transfer tube temperature; 275 °C; vaporizer temperature, 20 °C; MS^2^ detector, ion trap; scan rate, enhanced; MS^2^ AGC target, 10,000; MS^2^ maximum injection time, 100 ms; RF lens, 60%.

All accompanying resolution-of-identity (RI) Møller–Plesset perturbation theory/second-order algebraic diagrammatic construction [RI-MP2/ADC(2)] calculations were carried out using TURBOMOLE (v7.4.0).^45−47^ RI-MP2/ADC(2) calculations^48−51^ employed the CC2 routines implemented in TURBOMOLE^51−55^ and used the frozen-core approximation; the 12, 13, and 14 lowest-energy core orbitals of CMA, CA, and FA, were frozen in all RI-MP2/ADC(2) calculations. A tightened SCF convergence criterion of 1.0 × 10^–8^ au was used in all calculations; tightened convergence criteria of 1.0 × 10^–6^ and 3.0 × 10^–5^ au were used for the energy change and RMS gradient, respectively, in all geometry optimizations. The proper convergence of all geometry optimizations to real minima was verified via vibrational frequency inspection.

Where required, solvation effects were accounted for using the conductor-like screening model (COSMO) formalism.^56^ For single-point energy calculations, the iterative PTED (perturbation on energy and density) scheme was used to establish self-consistent COSMO reaction fields. For geometry optimizations, the PTED0 scheme was used; i.e., COSMO reaction fields were integrated into the solution of the CPHF equations for the relaxed densities via the PTE (perturbation on energy) scheme.^57−60^ The aug-cc-pVDZ basis set of Dunning et al.^61,62^ was used throughout and coupled with the correlate aug-cc-pVDZ/C auxiliary basis set.

## 3 Results and Discussion

### 3.1 *Ab Initio* Wave Function Theory Calculations of the Deprotomers of CMA, CA, and FA

High-level *ab initio* wave function theory calculations at the RI-MP2/ADC(2)/aug-cc-pVDZ level were performed on the selected coumaric acids in the gas phase and solution (EtOH and MeCN) to assign the experimental spectra. A number of minimum-energy deprotomer structures were identified for each deprotonated coumaric acid, both for the isolated (gaseous) and solution-phase environments. Table 1 lists the zero-point-corrected gas- and solution-phase energies obtained, with the corresponding structures and geometric parameters included in the Supporting Information (Scheme S1 and Tables S1–S9).

**Table 1:** Summary of Gas- and Solution-Phase (EtOH and MeCN) Energies (*E*_Rel._; Relative to the Most Stable Carboxylate Deprotomer for Each Environment and Each of CMA, CA, and FA), Vertical Detachment Energies (VDEs), and Vertical Dipole Moments (VDMs) for the CMA, CA, and FA Deprotomers^a^

	deprotomer	gas-phase *E*_Rel_(kJ mol^*–*1^)	VDE (eV)^b^	VDM (D)^c^	EtOH *E*_Rel_(kJ/mol^–1^)	MeCN *E*_Rel_(kJ/mol^–1^)
CMA	carboxylate	0.0	3.73	1.93	0.0	0.0
	phenoxide	–41.8	2.42	3.79	+16.3	+18.2
CA	carboxylate	0.0	3.74	3.04	0.0	0.0
	phenoxide (para)	–81.0	2.47	3.42	+0.4	+1.9
	phenoxide (meta)	–66.5	3.02	5.01	+5.7	+7.8
FA	carboxylate (*C*_*s*_)	0.00	3.70	3.64	0.0	N/A^d^
	carboxylate (*C*_1_)	+22.2	3.75	3.94	+14.7	0.0
	phenoxide (*C*_*s*_)	–26.6	2.23	2.70	+73.4	+61.2
	phenoxide (*C*_1_)	–32.8	2.42	2.39	+30.9	+17.6

a All energies are zero-point energy (ZPE) corrected. VDEs/VDMs are evaluated at the optimized gas-phase geometries. All values are evaluated at the RI-MP2/aug-cc-pVDZ level of theory.

b VDE = *E* (neutral at optimized anion geometry) – *E* (anion). This is included in the table for comparison with the experimental data.

c VDM is the dipole moment of the neutral at the geometry of the optimized anion geometry.

d Unobtainable as a stable minimum-energy geometry.

We found that the relative energies of the carboxylate and phenoxide deprotomers were strongly geometry-dependent^63^ and, consequently, dependent on the level of theory employed. This is challenging since accurate experimentally determined geometries are not available routinely for these anionic systems. For this reason, we focus here on the qualitative trends predicted by our calculations (Table 1). The calculations suggest that the phenoxide deprotomers exist at lower relative energies than the carboxylate deprotomers for the gaseous CMA, CA, and FA anions, but this trend inverts in solution (EtOH and MeCN), albeit with smaller energy differences between the two deprotomeric forms.

Given the results of the calculations, we anticipate that both carboxylate and phenoxide deprotomers of CMA and CA will be produced with approximately equal abundances upon ESI from solutions of EtOH and MeCN. More specifically, we predict carboxylate:phenoxide ratios of 1:1 and 1:2 for [CMA-H]^−^ and [CA-H]^−^, respectively. For [FA-H]^−^, we do not expect significant contributions from the *C*_*s*_-symmetric phenoxide deprotomer in EtOH or MeCN; this rotamer of the lower-energy *C*_1_-symmetric phenoxide deprotomer exists at considerably higher energy (+73.4 and +61.2 kJ mol^–1^ above the lowest-energy carboxylate deprotomer in EtOH and MeCN, respectively; Table 1). We predict that the remaining carboxylate and phenoxide deprotomers of [FA-H]^−^ produced from EtOH and MeCN occur with approximately abundances of 2:1 in EtOH and 1:1 in MeCN.

### 3.2 Gas-Phase UV–Vis Absorption Spectra of [CMA-H]^−^, [CA-H]^−^, and [FA-H]^−^

ESI readily produces the deprotonated forms of the coumaric acids [CMA-H]^−^, [CA-H]^−^, and [FA-H]^−^ as gas-phase ions with *m*/*z* 163, 179, and 193, respectively.

#### 3.2.1 [CMA-H]^−^

**Figure 1:**
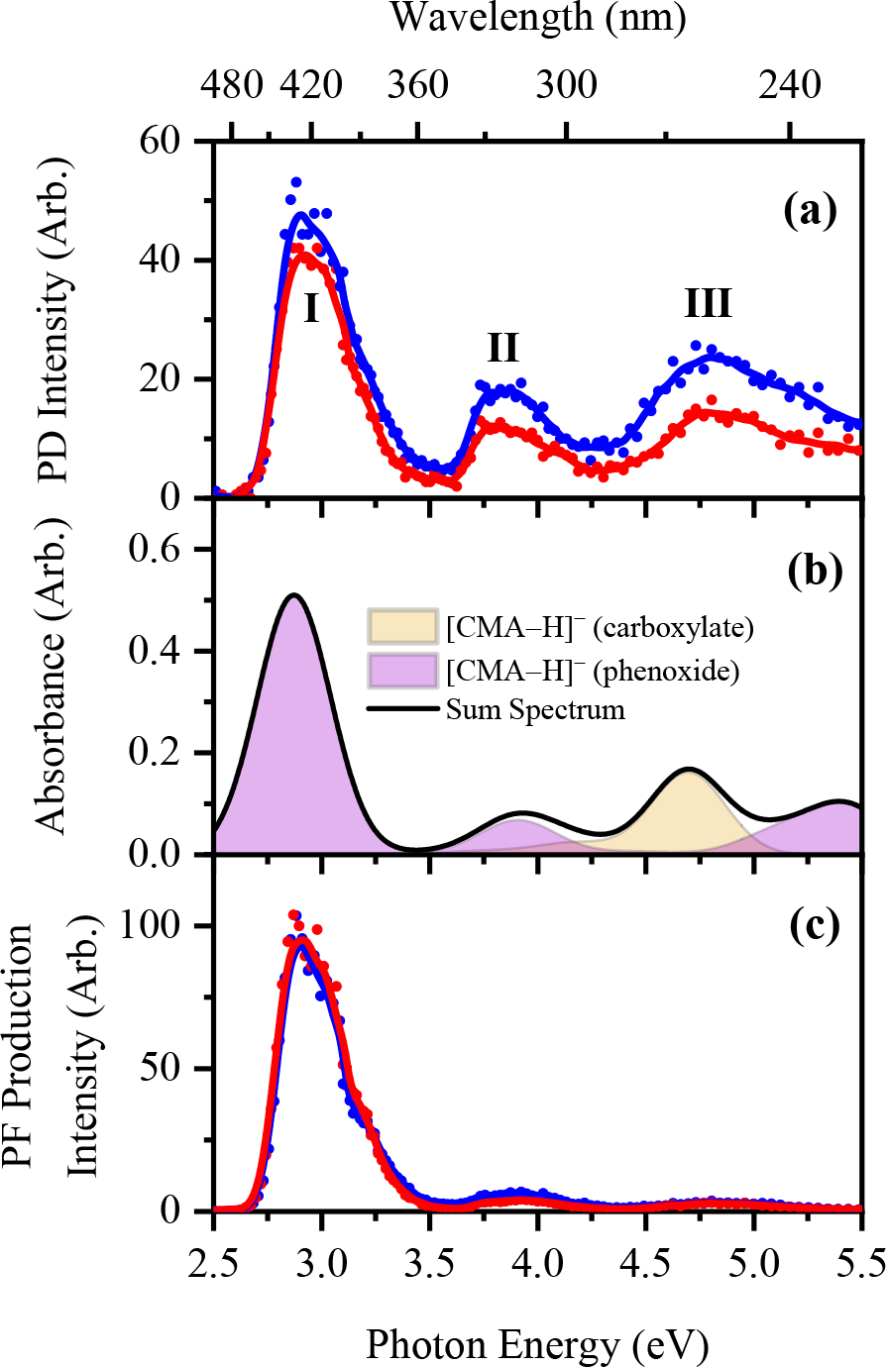
(a) Gas-phase photodepletion spectrum of [CMA-H]^−^ electrosprayed in EtOH (blue) and MeCN (red). (b) Theoretical gas-phase photoabsorption spectra of the carboxylate and phenoxide deprotomers of [CMA-H]^−^. (c) Photofragment production spectrum of the *m*/*z* 119 major photofragment of [CMA-H]^−^ produced when the anion is electrosprayed in EtOH (blue) and MeCN (red). The solid lines in (a) and (c) are five-point adjacent averages of the data points.

Figure 1a displays the gas-phase photodepletion (PD) spectra of [CMA-H]^−^ across the 2.5–5.5 eV (496–224 nm) spectral range. The solid blue and red lines represent the data obtained when electrospraying from EtOH and MeCN, respectively. Three distinct bands (**I**–**III**) are observed; a bright band (**I**) extending across the 2.6–3.4 eV region and two higher-energy absorption bands (**II**–**III**) peaking at ca. 3.8 and 4.8 eV, respectively. Notably, all three bands (**I**–**III**) appear when the anion is electrosprayed from both EtOH and MeCN.

Figure 1b displays the theoretical gas-phase absorption spectrum of [CMA-H]^−^ obtained at the RI-MP2/ADC(2)/aug-cc-pVDZ level. The sum spectrum (black line) was constructed using a 1:1 ratio (i.e., as an equally weighted linear combination) of the two deprotomers of [CMA-H]^−^. Comparison of the experimental and theoretical spectra clearly shows that both deprotomers must be present in the gas-phase ion ensemble, given that the simulated spectra in the band **I** and **III** regions in particular require contributions from both deprotomers. This is in line with the predicted relative energies of [CMA-H]^−^ (Table 1). Band **I** (λ_max_ ∼ 2.9 eV) in the experimental PD spectrum can be unambiguously assigned to a bright π* ← π transition from the phenoxide deprotomer (A′, 2.87 eV), while bands **II** and **III** were assigned to π* ← π transitions of the phenoxide (A′, 3.90 eV) and carboxylate (A′, 4.70 eV) deprotomers. Oomens and co-workers have previously used *in situ* IR spectroscopy of electrosprayed [CMA-H]^−^ to identify its deprotomers and identified similar ratios of carboxylate:phenoxide isomers as we observe here.^3^

We next turn to exploring the photofragment ions that are associated with excited state decay. [CMA-H]^−^ produces a single, dominant ionic photofragment at *m*/*z* 119 (Figure 1c) corresponding to the loss of *m*/*z* 44 (−CO_2_) from the parent ion. Oomens and co-workers were able to use *in situ* IR spectroscopy to identify this fragment as the *para*-vinylphenoxide anion.^3^ The production profile of the *m*/*z* 119 photofragment peaks at ca. 2.9 eV (eq 3a). Table 2 lists the ionic *m*/*z* of the major ionic photofragments of the deprotonated coumaric acids, along with their accompanying neutral fragments. The *m*/*z* 119 photofragment was observed following excitation of [CMA-H]^−^ produced via electrospray from both EtOH and MeCN.
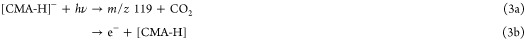
3a

**Table 2:** Summary of the Major Ionic Fragments of Electrosprayed [CMA-H]^−^, [CA-H]^−^, and [FA-H]^−^ Produced upon Higher-Energy Collisional Dissociation (HCD) and UV–Vis Laser Photoexcitation at *ca*. 2.95 and 4.75 eV

				obsd in laser photoexcitation (from EtOH ESI)^b^		obsd in laser photoexcitation (from MeCN ESI)^b^	
	ionic fragment*m*/*z*^a^	accompanying neutral fragment	obsd in HCD^b^	2.95 eV	4.75 eV	2.95 eV	4.75 eV
[CMA-H]^−^ (*m*/*z* 163)	119	CO_2_	√ (vs)	√ (vs)	√ (vw)	√ (vs)	√ (vw)
[CA-H]^−^ (*m*/*z*179)	135	CO_2_	√ (s)	√ (vs)	√ (vw)	√ (vs)	√ (vw)
	134	CO_2_ + H^•^	√ (w)	√ (w)		√ (w)	
[FA-H]^−^ (*m*/*z*193)	178	CH_3_^•^	√ (m)	√ (w)	√ (vw)	√ (m)	√ (vw)
	149	CO_2_	√ (w)	√ (m)		√ (s)	
	134	CH_3_^•^ + CO_2_	√ (s)	√ (w)	√ (s)	√ (m)	√ (vw)
	117		√ (vw)		√ (vw)		

a Determined with mass accuracy >0.3 amu.

b Very strong (vs), strong (s), medium (m), weak (w), and very weak (vw). Proposed structures are outlined in Table S10.

Additional minor photofragments of [CMA-H]^−^ were observed for the anions produced from electrospray in EtOH at *m*/*z* 121, 117, and 93 (Figure S7) and from electrospray in MeCN at *m*/*z* 145, 117, 93, and 91 (Figure S10). The minor photofragments can only be clearly observed through the band **I** region, likely due to electron detachment competing more effectively against ionic fragmentation as excitation energy increases. The VDE of the carboxylate and phenoxide deprotomers of CMA are calculated to be 3.73 and 2.42 eV (Table 1), respectively, so any electronic excitations lying above this energy occur within the electron detachment continuum. The propensity for electron detachment versus photofragmentation is discussed further in Section 3.3.

#### 3.2.2 [CA-H]^−^

**Figure 2:**
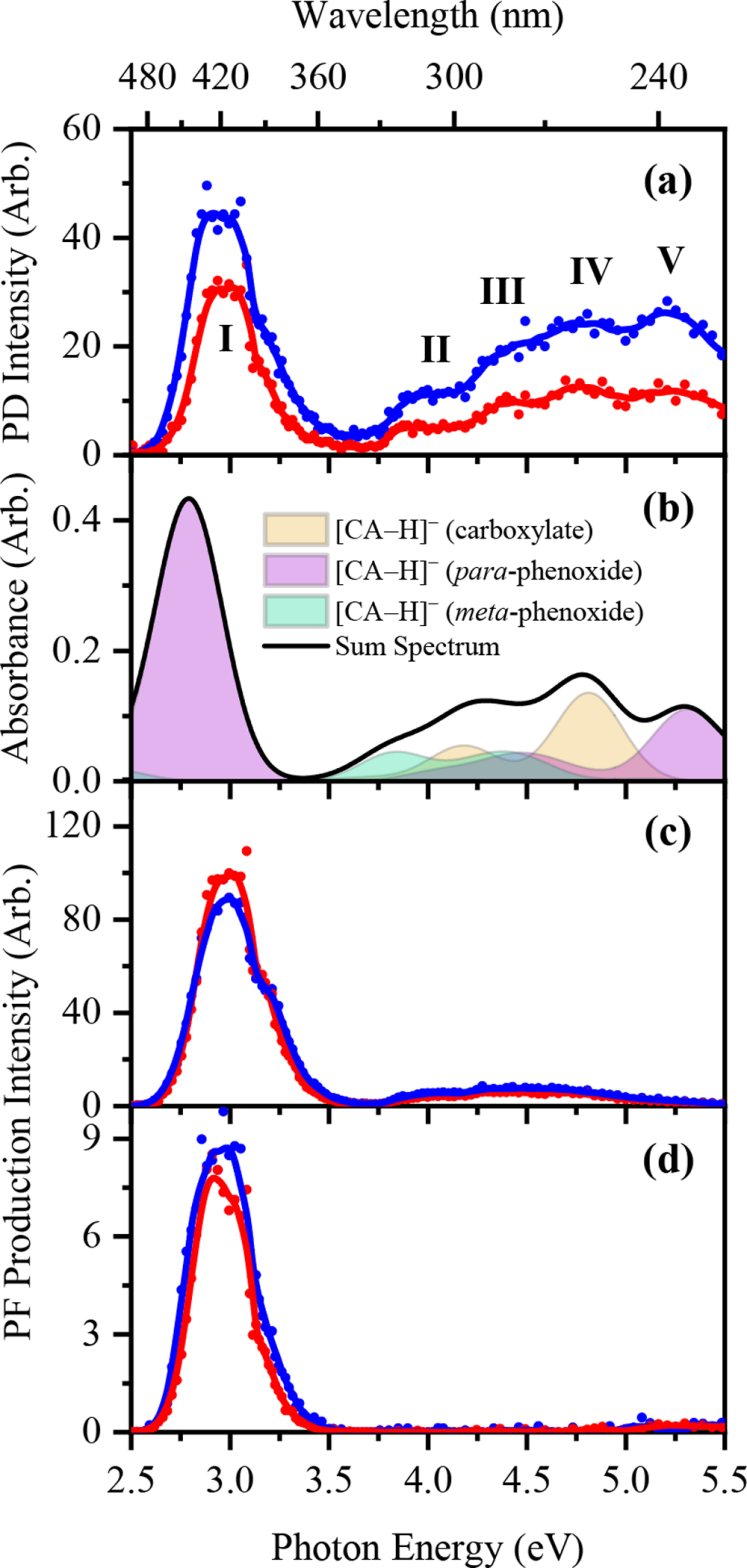
(a) Gas-phase photodepletion spectrum of [CA-H]^−^ as electrosprayed in EtOH (blue) and MeCN (red). (b) Theoretical gas-phase photoabsorption spectra of the carboxylate and phenoxide deprotomers of [CA-H]^−^. (c, d) Photofragment production spectrum of the only major photofragment of [CA-H]^−^ at *m*/*z* 135 and 134, respectively, as electrosprayed in EtOH (blue) and MeCN (red). The solid lines in (a), (c), and (d) are five-point adjacent averages of the data points.

Figure 2a displays the UV–vis laser PD spectrum of [CA-H]^−^ electrosprayed from EtOH and MeCN. Five broad absorption bands (**I**–**V**) are observed, with an initial strong feature (**I**) between 2.6 and 3.4 eV, followed by a cluster of somewhat less intense bands (**II**–**V**) from the UVB/UVC regions that peak at ca. 4.0, 4.4, 4.8, and 5.2 eV, respectively.

The theoretical gas-phase absorption spectrum of [CA-H]^−^ is displayed in Figure 2b (black line) and was constructed using a 1:2 carboxylate:phenoxide weighted linear combination of the spectral contributions for the two deprotomers. Band **I** corresponds to a bright π* ← π transition (A′, 2.79 eV) of the *para*-phenoxide deprotomer, clearly revealing the presence of the phenoxide deprotomer in the experimental spectrum. The somewhat broader bands (**II**–**V**) are more challenging to assign unambiguously, but bands **II** and **III** appear to result from the overlap of a number of bright π* ← π transitions originating from the carboxylate and *para*- and *meta*-phenoxide deprotomers, while band **IV** appears to be dominated by excitations of the carboxylate deprotomer (A′, 4.81 eV). (We used a ratio of 5:1 for the para:meta conformers to obtain the best fit to the experimental spectrum. It should be noted that these conformers can interconvert via proton transfer, so likely equilibrate with the more stable para-conformer then dominating.) Comparison of the experimental spectra obtained by electrospraying CA from both EtOH and MeCN with the theoretical summed spectrum clearly shows that the experimental spectrum must result from both isomers being present in the electrosprayed ion ensemble in the ratio of ∼1:2 carboxylate:phenoxide.

Photoexcitation of [CA-H]^−^ is associated with two significant ionic photofragmentation channels (eqs 4a and 4b) that correspond to the production of the *m*/*z* 135 and 134 photofragments (Figures 2b and 2c; Table 2) associated with loss of neutral CO_2_ and H + CO_2_, respectively. Both of these photofragments are produced predominantly through the band **I** region, corresponding to photoexcitation of the lowest-lying singlet excited state of the *para*-phenoxide deprotomer. Relatively minor photofragments of [CA-H]^−^ can be observed at *m*/*z* 133, 117, 109, 107, and 91 for electrospray from EtOH and at *m*/*z* 133, 117, 109, and 107 for electrospray from MeCN (Figures S8 and S11).
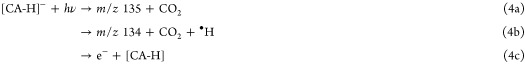
4a

The propensity for electron detachment versus photofragmentation is discussed further in Section 3.3.

#### 3.2.3 [FA-H]^−^

**Figure 3:**
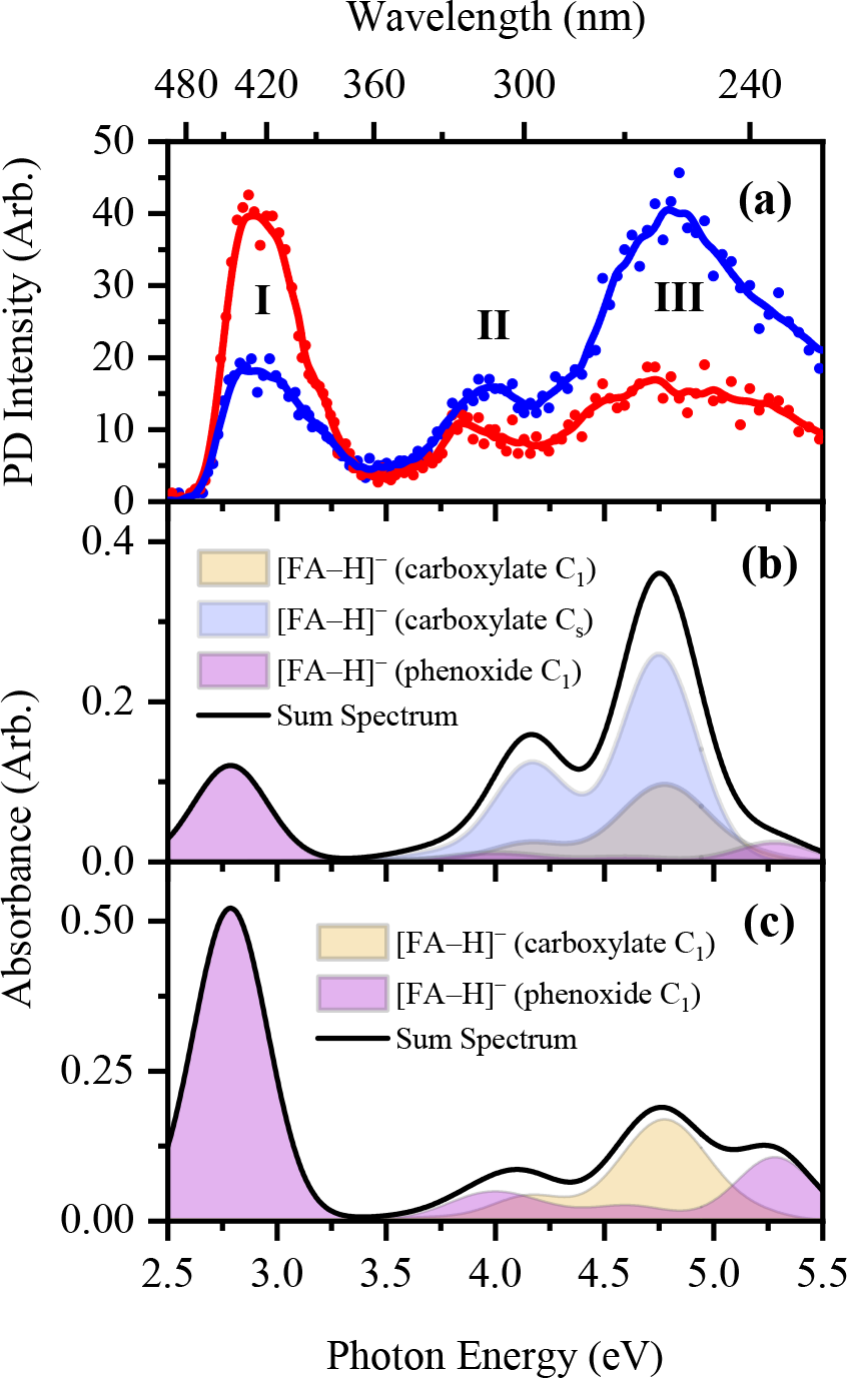
(a) Gas-phase photodepletion spectrum of [FA-H]^−^ electrosprayed in EtOH (blue) and MeCN (red). The solid line is a five-point adjacent average of the data points. Theoretical spectra of the addressed carboxylate and phenoxide deprotomer(s) of [FA-H]^−^ as determined for (b) EtOH and (c) MeCN, respectively.

Figure 3a displays the gas-phase UV–vis PD spectra of [FA-H]^−^. In contrast to that of [CMA-H]^−^ and [CA-H]^−^, the PD spectrum of [FA-H]^−^ changes considerably depending on whether FA is electrosprayed in EtOH or MeCN. Using EtOH as the solvent (blue spectrum), we observe three absorption features (labeled **I**–**III**) with an absorption onset at the lowest energy band (**I**) of 2.6 eV. This band relatively weak and broad and is observed to extend from the visible region into the UV (2.6–3.4 eV). A similarly broad band (**II**) is observed between 3.4 and 4.25 eV, with a maximum at ca. 4.0 eV. Band **III** appears as a rather strong feature with an absorption extending between 4.3 and 5.5 eV and peaking prominently at 4.8 eV. In contrast when FA is electrosprayed from MeCN (red spectrum), band **I** dominates the PD spectrum of [FA-H]^−^, while bands **II** and **III** are significantly less intense.

In [CMA-H]^−^ and [CA-H]^−^, band **I** was unambiguously assigned to a bright π* ← π transition originating from a phenoxide deprotomer, with the higher-energy bands being assigned to π* ← π transitions originating from carboxylate deprotomers. Our calculations predict the same assignments for [FA-H]^−^; band **I** arises from the bright π* ← π transition of the *C*_1_-symmetric phenoxide deprotomer (A′, 2.79 eV), while bands **II** and **III** are associated with transitions of the carboxylate deprotomers. The greater changes observed for the PD spectra upon electrospraying from EtOH and MeCN for FA reveal that production of deprotomers for this molecule is a more sensitive function of solvent than for CMA and CA. (The distribution of deprotomers will be affected by the kinetic process of electrospray and can be affected by the formation of dimers during transfer of the molecules from solution to the gas phase. This formation of such dimers is clearly a function of whether the electrospray solvent is protic or aprotic. These effects are discussed in more detail in refs (3) and (8).) Figures 3b and 3c display the theoretical gas-phase absorption spectra of [FA-H]^−^ with a 2:1 and 1:1 carboxylate:phenoxide ratio providing a good match to the experimental spectra.

Photofragmentation of [FA-H]^−^ produced from electrospray of FA in both solvents produces *m*/*z* 178, 149, and 134 as the dominant ionic products (eqs 5a–5d), corresponding to the loss of mass units 15 (−[CH_3_]^•^), 44 (−CO_2_), and 59 (−[CH_3_]^•^ + −CO_2_) from the parent ion (Table 2). A number of relatively minor photofragments can also be observed at *m*/*z* 137, 133, 119, 117, 108, and 89 when electrospray occurs from EtOH (Figure S9). The same minor photofragments were observed when [FA-H]^−^ was produced from MeCN electrospray.
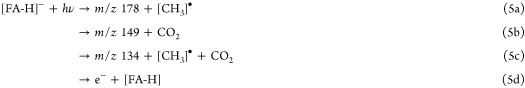
5a

We note that pathways 5a and 5c both result in the production of free radical species. Similar free radical products have been observed for the deprotonated forms of the sunscreens oxybenzone, 2-phenylbenzimidazole-5-sulfonic acid, and benzophenone-4 in recent work.^36−38^

**Figure 4:**
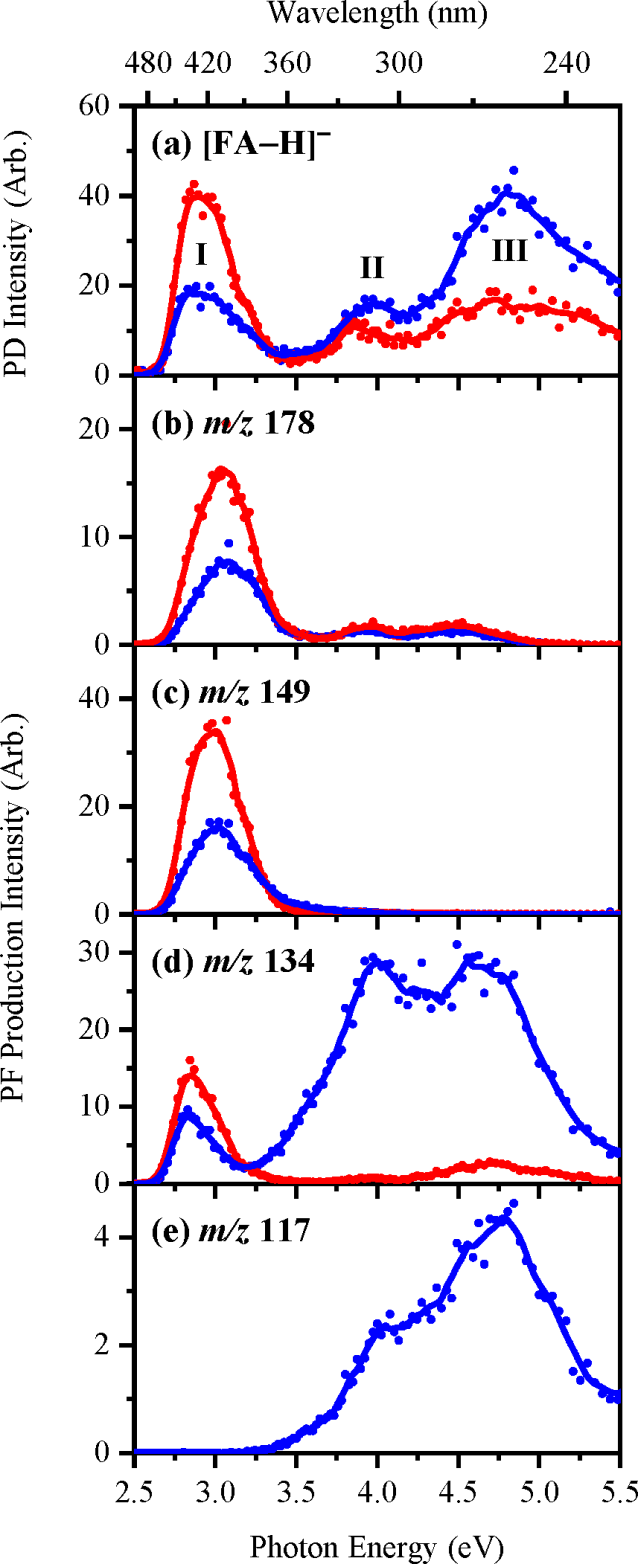
(a) Gas-phase photodepletion spectrum of [FA-H]^−^ electrosprayed in EtOH (blue) and MeCN (red). (b–e) Photofragment production spectra of the four major photofragments of [FA-H]^−^ with *m*/*z* 178, 149, 134, and 117, respectively, as electrosprayed in EtOH (blue) and MeCN (red). The solid lines are a five-point adjacent average of the data points.

Figures 4b–4e present the action spectra for the four major ionic photofragments of [FA-H]^−^ electrosprayed from EtOH (blue spectrum). The precursor [FA-H]^−^ (*m*/*z* 193) PD spectrum is reproduced in Figure 4a to facilitate comparison. It is evident from Figure 4 that the relative production yield of the four major photofragments of [FA-H]^−^ at *m*/*z* 178, 149, 134, and 117 varies significantly as a function of photon energy. The production spectra of *m*/*z* 178 and 149 (Figures 4b and 4c), respectively, show that both photofragments are primarily produced across the 2.6–3.6 eV (visible-UVA) region, with *m*/*z* 178 produced only in low yield around 4.0 and 4.6 eV. The action spectrum of *m*/*z* 134 (Figure 4d), however, shows that the fragment is produced across the entire spectral range with production peaking close to the band maxima **I**–**III** (Figure 4a). We note that production of *m*/*z* 134 is enhanced through band **II**. The production of the photofragment at *m*/*z* 117 (Figure 4e) is almost 10-fold less intense than the *m*/*z* 149 and 134 ions and is produced only through bands **II** and **III**.

The photofragment production spectra obtained from [FA-H]^−^ when it is electrosprayed from MeCN are presented in red on Figures 4b–4e. Electrospraying from MeCN dramatically reduced ionic fragment production at *ca*. 3.5 eV, with the *m*/*z* 134 photofragment ion (the major photofragment when FA is electrosprayed from MeCN) almost entirely absent.

Figure S12 presents the relative ion yield spectra of the photofragments of [FA-H]^−^ electrosprayed from both EtOH and MeCN, providing an overview of their relative production. These plots show that production of *m*/*z* 149, which corresponds to the loss of CO_2_ from [FA-H]^−^, is strongly enhanced through the band **I** region, which is associated with the phenoxide deprotomer. The *m*/*z* 134 photofragment (eq 5c) shows a generally increasing trend in production with photon energy, although the resulting production profile does vary for electrospray from the two solvents. Production of *m*/*z* 178 can be seen to decrease as production of *m*/*z* 134 increases, suggesting that the *m*/*z* 134 ion may be a secondary photofragment produced when *m*/*z* 178 has high internal energy.

The propensity for electron detachment versus photofragmentation is discussed further in the next Section 3.3.

### 3.3 Electron Detachment Yield vs Photodepletion Yield Interpretation

**Figure 5:**
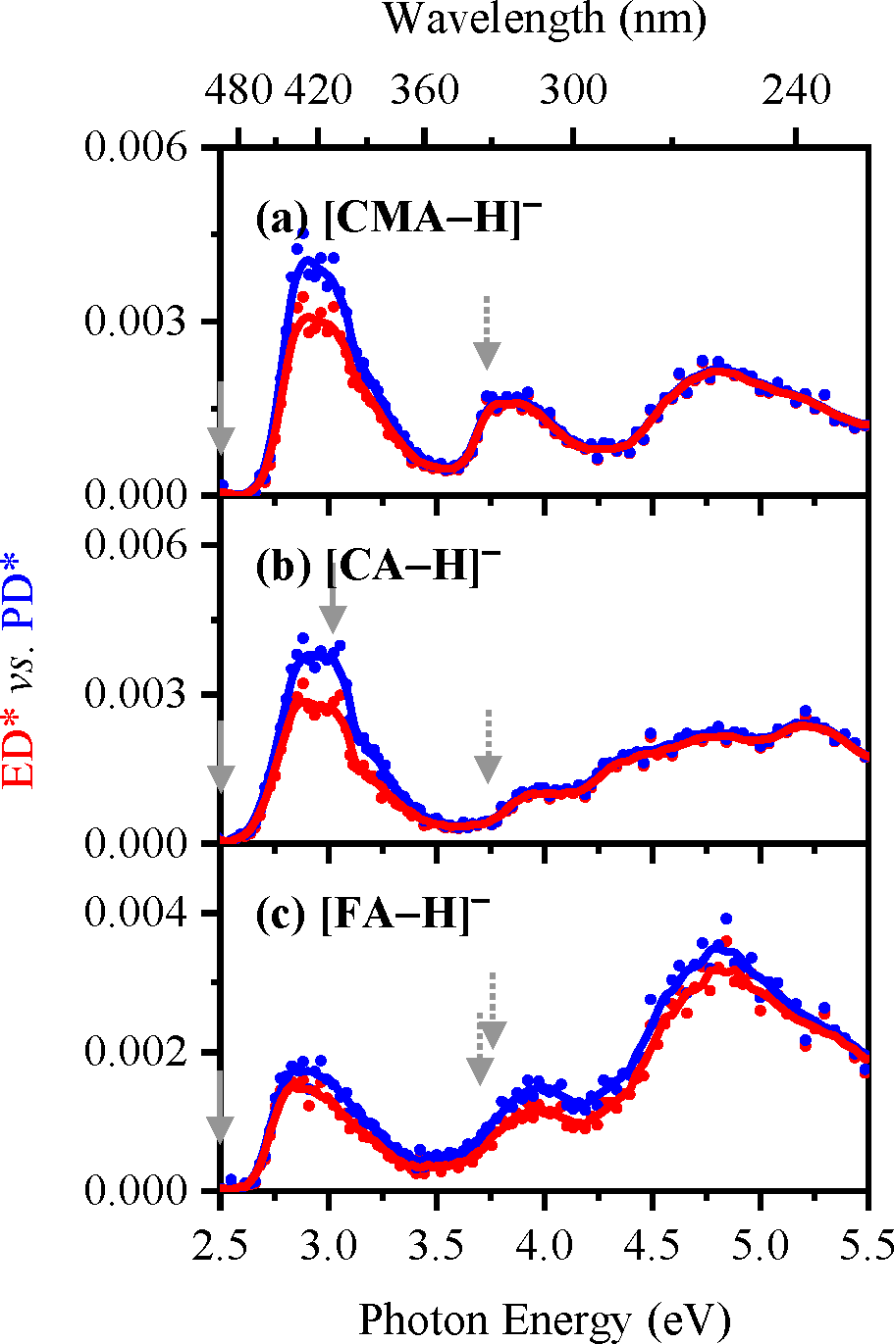
Electron detachment yield (ED*; red) vs photodepletion yield (PD*; blue) spectra of (a) [CMA-H]^−^, (b) [CA-H]^−^, and (c) [FA-H]^−^ when electrosprayed in EtOH, respectively. The solid lines are a five-point adjacent average of the data points. The overlaid arrows represent the calculated VDEs of the phenoxide (solid gray) and carboxylate (dotted gray) deprotomers, as outlined in Table 1.

**Figure 6:**
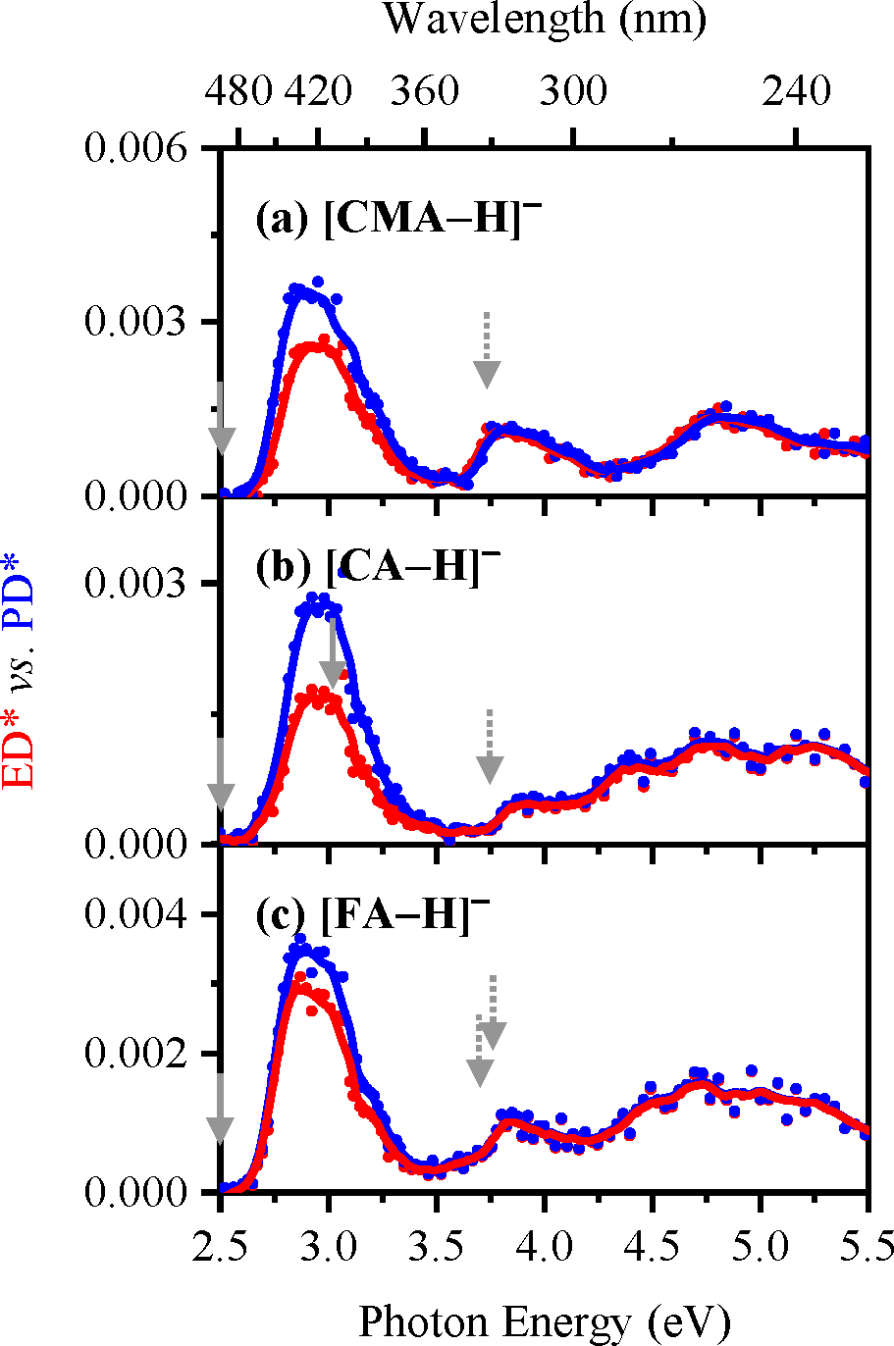
Electron detachment yield (ED*; red) vs photodepletion yield (PD*; blue) spectra of (a) [CMA-H]^−^, (b) [CA-H]^−^, and (c) [FA-H]^−^ when electrosprayed in MeCN, respectively. The solid lines are a five-point adjacent average of the data points. The overlaid arrows represent the calculated VDEs of the phenoxide (solid gray) and carboxylate (dotted gray) deprotomers, as outlined in Table 1.

The electron detachment yield spectra of [CMA-H]^−^, [CA-H]^−^, and [FA-H]^−^ are displayed in Figures 5a–5c and 6, with the corresponding photodepletion spectra for comparison. Electron loss is not directly measurable in our instrument, so these spectra are obtained by assuming that any photodepleted ions not detected as ionic photofragments are lost through electron detachment. (This is true for the situation where ionic fragments with *m*/*z* < 50 represent only minor decay pathways.) Further discussion of electron detachment yield spectra can be found in ref.^42^^42^

Figures 5a–5c present the electron detachment yield spectra obtained when electrospraying in ethanol, while Figures 6a–6c display spectra obtained when the solvent is acetonitrile. For both sets of spectra, the electron detachment yield curves display very similar profiles to the corresponding photodepletion spectra, indicating that electron detachment is the main photodepletion pathway. Ionic fragmentation can be seen to be relatively more significant through the band **I** regions (2.5–3.25 eV), with electron detachment becoming increasingly more dominant as the photoexcitation energy increases. We note that the calculated VDEs for the phenoxide and carboxylate isomers of the [CMA-H]^−^, [CA-H]^−^, and [FA-H]^−^ anions are predicted to be around 2.4 and 3.7 eV, respectively (Table 1). The entire photodepletion region scanned here therefore lies at or above the detachment energies for the phenoxide deprotomers, with the band **II** and **II** regions lying above the VDEs of the carboxylate isomers. Despite the fact that the photodepletion spectra are being acquired above the detachment energies, the electronic absorptions are clearly mapped on the electron detachment continua, as we have observed in other systems.^64,65^

### 3.4 Higher-Energy Collisional Dissociation vs Photofragmentation

**Figure 7:**
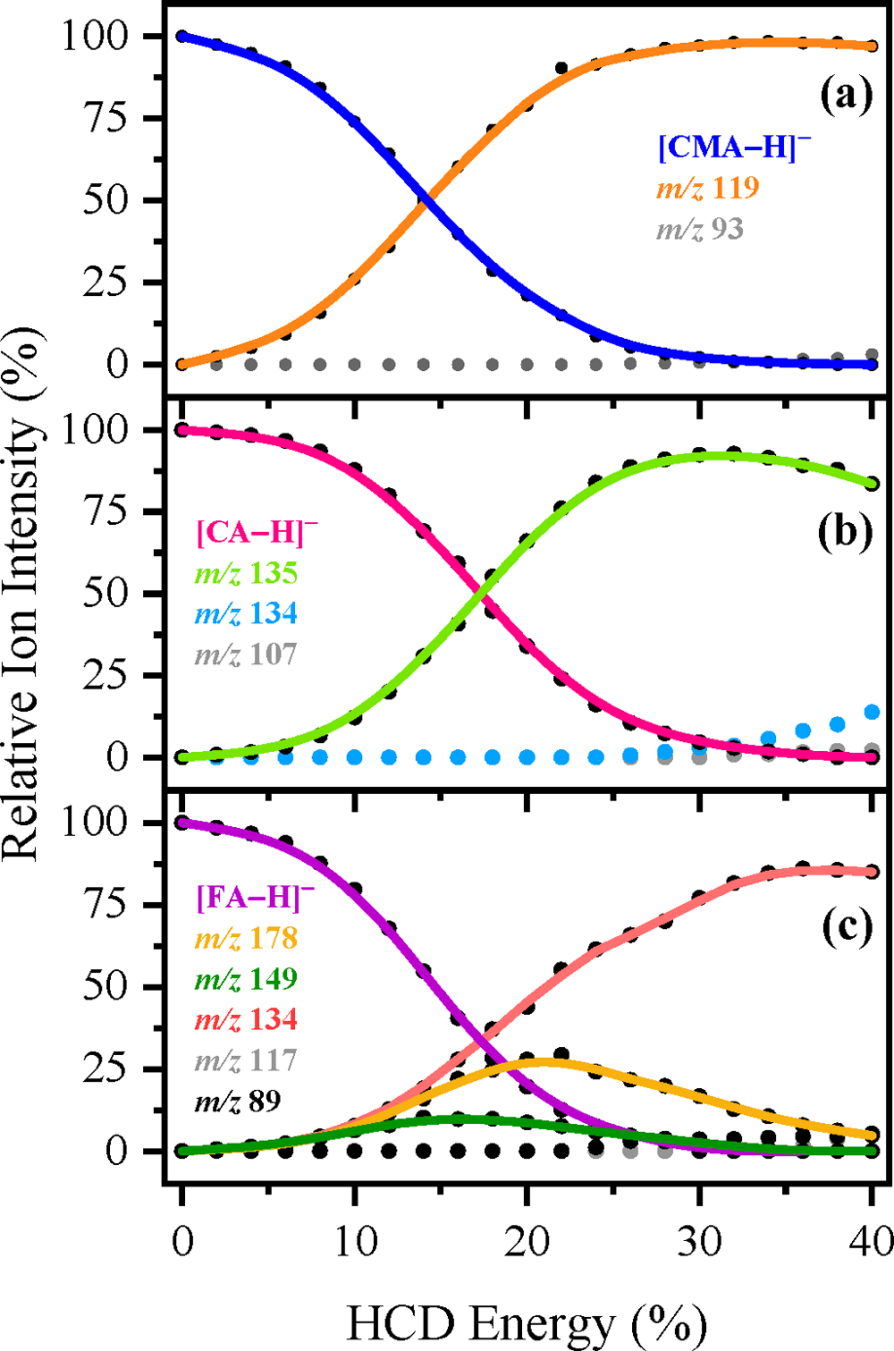
Parent ion dissociation curves for solutions of (a) [CMA-H]^−^ (*m*/*z* 163), (b) [CA-H]^−^ (*m*/*z* 179), and (c) [FA-H]^−^ (*m*/*z* 193) electrosprayed in EtOH, respectively, for their most intense ionic fragments formed upon HCD between 0 and 40% energies. The curved lines are a three-point adjacent average of such data points and are provided as a viewing guide, to emphasize the profile for each individual fragment.

**Figure 8:**
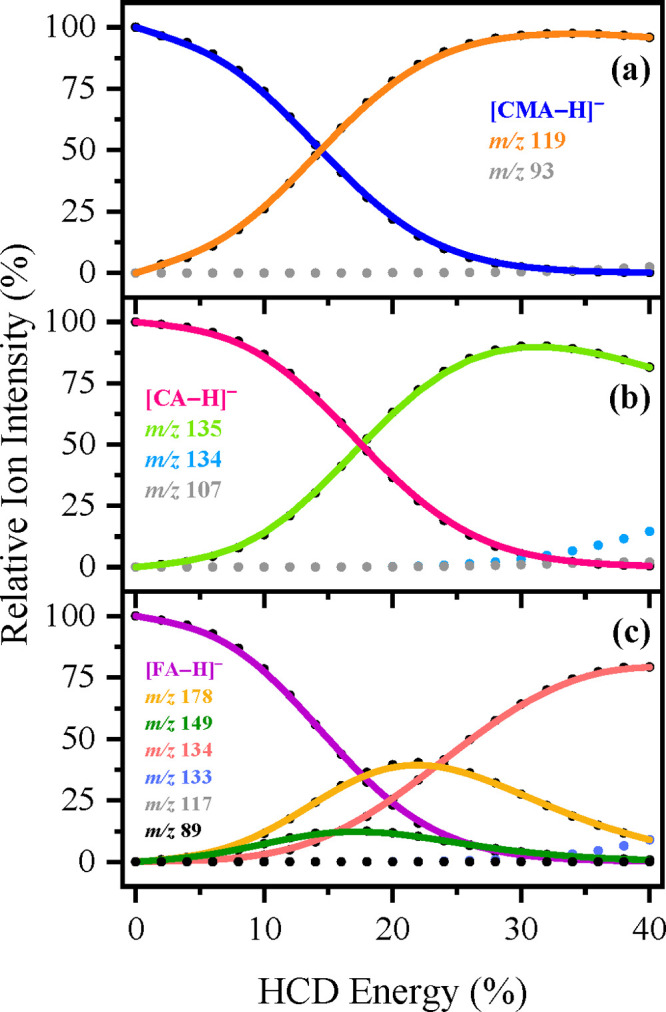
Parent ion dissociation curves for solutions of (a) [CMA-H]^−^ (*m*/*z* 163), (b) [CA-H]^−^ (*m*/*z* 179), and (c) [FA-H]^−^ (*m*/*z* 193) electrosprayed in MeCN, respectively, for their most intense ionic fragments formed upon HCD between 0 and 40% energies. The curved lines are a three-point adjacent average of such data points and are provided as a viewing guide, to emphasize the profile for each individual fragment.

To investigate the thermal fragmentation pathways of the deprotonated antioxidants on their electronic ground states, higher-energy collisional dissociation (HCD) was employed (Figures 7a–7c and 8a–8c; Table 2). These measurements are essential to identify ions that are secondary fragments, i.e., ionic fragments that are formed when a precursor species breaks apart at high internal energy.^38,43^ (ref (43) gives a full discussion of how the HCD energies can be related to ion internal energies obtained upon photoexcitation.) In addition, these measurements are important as any photofragments not observed in HCD studies can be identified as purely photochemical products.

Collisional activation of [CMA-H]^−^ produced from both EtOH and MeCN (Figures 7a and 8a, respectively), produces a single major HCD product with *m*/*z* 119. (A *m*/*z* 93 ion is produced as an extremely minor fragment at the very highest collisional energies measured.) The *m*/*z* 119 ion is the only significant photofragment observed for [CMA-H]^−^.

For [CA-H]^−^ electrosprayed from both EtOH and MeCN (Figures 7b and 8b), HCD produces the *m*/*z* 135 ion (loss of CO_2_) as the dominant fragment. The ion at *m*/*z* 134 is observed as a very low intensity fragment >30% HCD energy. *m*/*z* 135 and 134 are the major and minor photofragments of [CA-H]^−^, and our HCD results suggest that *m*/*z* 134 photofragment is a secondary photofragment produced at high internal energies of *m*/*z* 135 (since *m*/*z* 134 increases over the same energy range that *m*/*z* 135 decreases). For both [CMA-H]^−^ and [CA-H]^−^, the similar profile of HCD fragment production when the deprotonated molecule is electrosprayed from either EtOH or MeCN indicates that the proportion of deprotomers present following electrospray is broadly similar when either solvent is used.

Collisional activation of [FA-H]^−^ from both EtOH and MeCN (Figures 7c and 8c) results in the *m*/*z* 134 fragment (loss of [CH_3_]^•^ + CO_2_) as the dominant product, with *m*/*z* 178 (loss of [CH_3_]^•^) and 149 (loss of CO_2_) appearing as medium intensity fragments. The intensity of both *m*/*z* 178 and 149 decreases at higher collisional energies, indicating that *m*/*z* 178 is fragmenting into *m*/*z* 134 at higher internal energy. (*m*/*z* 117 and 89 are observed as extremely minor fragments >35% HCD energy.) Notably, there is a significant difference in the relative ion production intensities of the *m*/*z* 178 and 134 fragments from [FA-H]^−^ for electrospray from the two solvents. When EtOH is employed as the electrospray solvent, *m*/*z* 134 dominates as the most intense ion across the HCD energies scanned (e.g., at 17.4% HCD energy: *m*/*z* 134 = 34% and *m*/*z* 178 = 23%). In contrast, when MeCN is used as the electrospray solvent, *m*/*z* 178 is the main ion observed between 0 and 24% HCD energy (e.g., at 17.4% HCD energy: *m*/*z* 134 = 17% and *m*/*z* 178 = 34%). This is consistent with the proportion of the two possible deprotomers varying when different electrospray solvents are employed.

Finally, we note that the major HCD fragments mirror the major gas-phase photofragments, which suggests that these deprotonated coumaric acids are relaxing by predominantly statistical (ergodic) decay over the spectral range (2.5–5.5 eV) studied.^38^

## 4 Conclusions

Recent time-resolved laser spectroscopic and theoretical studies of the CMA, CA, and FA have explored their photodynamics,^20−22,27,28,32,33^ revealing that the molecules can undergo rapid nonradiative excited state decay via a *cis*–*trans* isomerization process.^28^ A limited number of studies have explored how these photodynamics are affected by pH and, hence, the extent of deprotonation, finding that deprotonation had little effect on excited state decay.^27^ This result is consistent with our results here for the deprotonated coumaric acids, since the ionic fragmentation patterns for UV–vis laser and HCD excitation are consistent with statistical fragmentation which is associated with rapid ultrafast decay.^38^ This is perhaps not surprising given that the coumaric acid deprotonation sites are not the key parts of the molecule that effect the nonradiative transition from the excited state back to the electronic ground state.^28^ However, the results presented here provide confirmation that the action of coumaric acid based antioxidants are likely to perform well as UV filters even in mildly alkaline environments such as seawater.

Two recent studies have used a custom-built ion mobility mass spectrometer to study deprotonated coumaric acids formed via electrospray.^66,67^ The main focus of these studies was on the photoisomerisation dynamics and excited state proton transfer. Carboxylate and phenoxide deprotomers were identified in these studies through their differential collisional cross sections. Ion mobility separation of deprotomers will be advantageous for systems where the optical transitions associated with a pair of deprotomers are not well separated. While ion mobility detection is clearly complementary to the UV–vis laser photodissociation spectroscopy within the commercial mass spectrometer presented here, it is important to have a range of approaches available since what is crucial is that they can be applied in situ to identify deprotomeric species. This is of particular importance for the reaction studies discussed in the introduction, where the type of mass spectrometer available for ESI detection may vary.^14−17^

The results presented above show that laser photodissociation spectroscopy within a laser-interfaced mass spectrometer combined with theoretical wave function calculations can identify the phenoxide and carboxylate deprotomers of coumaric acids produced by ESI. For deprotonated CA and CMA, we find that the ratio of phenoxide:carboxylate deprotomers is relatively insensitive to electrospray in both protic and aprotic solvents. The ratio of deprotomers of FA is considerably more strongly affected by the electrospray solvent employed, with electrospray from aprotic MeCN leading to a higher proportion of the carboxylate deprotomer, which is favored in solution. Overall, our results show unequivocally that a mixture of carboxylate:phenoxide deprotomers is formed upon electrospray for all three coumaric acids studied here. This leads us to conclude that this is likely to be a general phenomenon when deprotonated coumaric acids are produced via electrospray.^20,21^

While a number of studies have now used UV–vis laser photodissociation to identify protomers,^1,7,8,65^ to our knowledge our study represents the first where photodissociation supported by advanced quantum chemical calculations has been used to definitively identify deprotomeric ions. Given that there are a growing number of fundamental studies being conducted on gaseous ions produced via electrospray, as well as electrospray mass spectrometry being used as an analytic tool to monitor solution-phase reactions,^14−17^ it is important to have tools available to spectroscopically identify deprotomeric (or protomeric) structures. Although *in situ* IR spectroscopy has been used for this purpose,^3,11−13^ free electron lasers have typically been employed in these experiments, which leads to challenges due to access limitations. Our results demonstrate an accessible alternative *in situ* approach using adapted commercial instrumentation that can successfully determine deprotonation ratios in electrosprayed ions with UV chromophores.

## Supporting Information Available

Deprotomer structures of [CMA-H]^−^, [CA-H]^−^, and [FA-H]^−^, optimized Cartesian coordinate tables for [CMA-H]^−^, [CA-H]^−^, and [FA-H]^−^; photodepletion laser power dependence measurements; additional photofragment action spectra; an ion yield plot for [FA-H]^−^; proposed structures of the major ionic fragments; excited state assignments obtained from the calculations; key molecular orbitals (PDF)
