# Supplementary material for: Measurement of the Population of Electrosprayed Deprotomers of Coumaric Acids Using UV–Vis Laser Photodissociation Spectroscopy
Source: J Phys Chem A. 2021 Aug 3;125(31):6703–14. doi: 10.1021/acs.jpca.1c04880 (PMC8389988; doi:10.1021/acs.jpca.1c04880)
Supplement: Supplementary file 1 — jp1c04880_si_001.pdf [file jp1c04880_si_001.pdf]

Supporting Information

Measurement of the Population of Electrosprayed Deprotomers of Coumaric Acids using UV-Vis Laser Photodissociation Spectroscopy

Natalie G. K. Wong,[a] Conor D. Rankine,[b] and Caroline E. H. Dessent[a]*

[a] Department of Chemistry, University of York, Heslington, York, YO10 5DD, U.K.

[b] School of Natural and Environmental Sciences, Newcastle University, Newcastle-upon-Tyne, NE1 7RU, U.K.

* Corresponding author: Email: caroline.dessent@york.ac.uk

Supporting Information:

S1. Deprotomer structures of $[CMA–H]^–$, $[CA–H]^–$, and $[FA–H]^–$

S2. Optimized Cartesian coordinate tables

S3. Photodepletion laser power dependence measurements

S4. Additional photofragment action spectra

S5. Ion yield plot for $[FA–H]^–$

S6. Proposed structures of major ionic fragments

S7. Additional calculation details: Excited state assignments

S1. Deprotomer structures of [CMA–H]⁻, [CA–H]⁻, and [FA–H]⁻

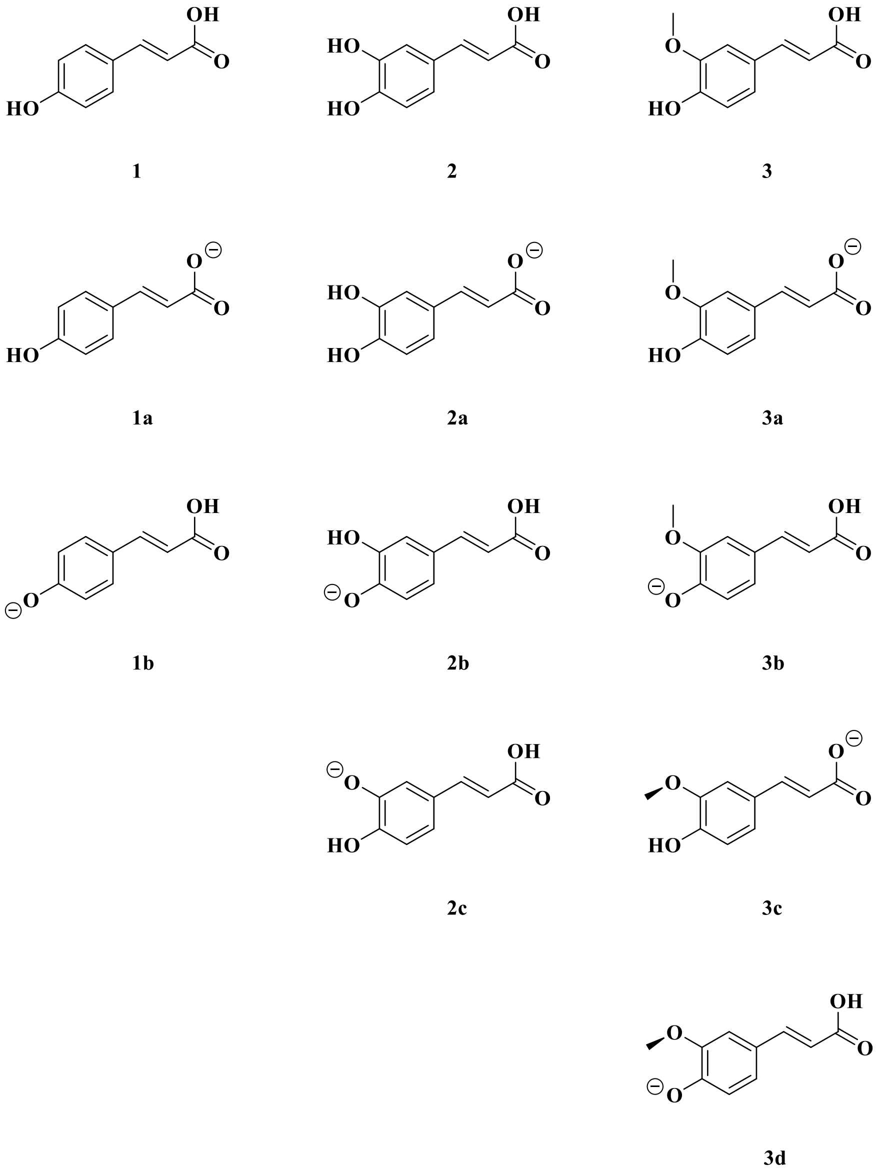

Scheme S1. Schematic highlighting the deprotomeric structures of the **(1)** [CMA–H]⁻: **(a)** carboxylate and **(b)** phenoxide isomer; **(2)** [CA–H]⁻: **(a)** carboxylate, **(b)** *para*-phenoxide, and **(c)** *meta*-phenoxide isomer; and **(3)** [FA–H]⁻: **(a)** carboxylate (C_s), **(b)** phenoxide (C_s), **(c)** carboxylate (C_1), and **(d)** phenoxide (C_1) isomer.

S2. Optimized Cartesian coordinate tables

Table S1. Optimized Cartesian coordinates in Å and ground-state RI-MP2 energies, $E_{\text{RI-MP2}}$, in atomic units (a.u.) for the minimum-energy geometry of $[\text{CMA–H}]^-$ (carboxylate isomer) at the RI-MP2/*aug*-cc-pVDZ level.

$E_{\text{RI-MP2}} = -571.3915610011$			
C	−0.209031	0.122539	0.000000
C	1.098951	−0.428118	0.000000
C	1.315886	−1.814535	0.000000
C	0.219757	−2.695523	0.000000
C	−1.088121	−2.183688	0.000000
C	−1.291361	−0.790905	0.000000
C	−0.483701	1.570109	0.000000
C	0.431028	2.575363	0.000000
C	0.037830	4.058694	0.000000
O	0.493436	−4.061785	0.000000
O	1.029861	4.856401	0.000000
O	−1.204740	4.316601	0.000000
H	1.962361	0.243204	0.000000
H	2.328948	−2.227554	0.000000
H	−1.946632	−2.866889	0.000000
H	−2.313196	−0.396527	0.000000
H	−1.536563	1.881872	0.000000
H	1.509523	2.369114	0.000000
H	−0.354236	−4.528375	0.000000

Table S2. Optimized Cartesian coordinates in Å and ground-state RI-MP2 energies, $E_{RI\text{-}MP2}$, in atomic units (a.u.) for the minimum-energy geometry of $[CMA–H]^{–}$ (phenoxide isomer) at the RI-MP2/*aug*-cc-pVDZ level.

$E_{RI\text{-}MP2}$ = −571.4081425663			
C	−0.138346	−0.370173	0.000000
C	1.162780	−0.962712	0.000000
C	1.332993	−2.341681	0.000000
C	0.212998	−3.279995	0.000000
C	−1.097837	−2.645314	0.000000
C	−1.254494	−1.263329	0.000000
C	−0.369831	1.044940	0.000000
C	0.562814	2.065065	0.000000
C	0.210517	3.468068	0.000000
O	0.375994	−4.541774	0.000000
O	0.999114	4.423773	0.000000
O	−1.160118	3.721703	0.000000
H	2.049131	−0.316503	0.000000
H	2.339954	−2.775966	0.000000
H	−1.972724	−3.306090	0.000000
H	−2.264568	−0.830680	0.000000
H	−1.425404	1.349307	0.000000
H	1.637396	1.868901	0.000000
H	−1.200372	4.692460	0.000000

Table S3. Optimized Cartesian coordinates in Å and ground-state RI-MP2 energies, $E_{\text{RI-MP2}}$, in atomic units (a.u.) for the minimum-energy geometry of $[\text{CA–H}]^-$ (carboxylate isomer) at the RI-MP2/*aug*-cc-pVDZ level.

$E_{\text{RI-MP2}} = -646.4623809060$			
C	0.331049	0.311807	0.000000
C	−0.936595	−0.324343	0.000000
C	−1.043528	−1.718664	0.000000
C	0.117842	−2.517451	0.000000
C	1.382586	−1.915741	0.000000
C	1.483817	−0.511601	0.000000
C	0.495267	1.775742	0.000000
C	−0.490000	2.711860	0.000000
C	−0.202057	4.218864	0.000000
O	−0.099970	−3.898632	0.000000
O	−1.247004	4.945855	0.000000
O	1.019805	4.563299	0.000000
O	−2.294391	−2.310528	0.000000
H	−1.857231	0.265448	0.000000
H	2.283774	−2.540994	0.000000
H	2.471568	−0.040445	0.000000
H	1.522341	2.163746	0.000000
H	−1.550890	2.428305	0.000000
H	0.762067	−4.336637	0.000000
H	−2.148452	−3.269891	0.000000

Table S4. Optimized Cartesian coordinates in Å and ground-state RI-MP2 energies, $E_{\text{RI-MP2}}$, in atomic units (a.u.) for the minimum-energy geometry of $[\text{CA–H}]^-$ (*para*-phenoxide isomer) at the RI-MP2/*aug*-cc-pVDZ level.

$E_{\text{RI-MP2}}$ = −646.4945342829			
C	0.381305	−0.186978	0.000000
C	−0.902039	−0.822225	0.000000
C	−0.974980	−2.207394	0.000000
C	0.197596	−3.076437	0.000000
C	1.465961	−2.407549	0.000000
C	1.544366	−1.010255	0.000000
C	0.549355	1.239717	0.000000
C	−0.427042	2.212556	0.000000
C	−0.064563	3.618418	0.000000
O	−0.013280	−4.346945	0.000000
O	−1.190846	4.440872	0.000000
O	1.060359	4.128298	0.000000
O	−2.160598	−2.894778	0.000000
H	−1.826604	−0.235538	0.000000
H	2.373237	−3.021864	0.000000
H	2.525746	−0.518800	0.000000
H	1.587002	1.603321	0.000000
H	−1.492506	1.973787	0.000000
H	−1.816247	−3.826228	0.000000
H	−0.816221	5.338022	0.000000

Table S5. Optimized Cartesian coordinates in Å and ground-state RI-MP2 energies, $E_{\text{RI-MP2}}$, in atomic units (a.u.) for the minimum-energy geometry of $[\text{CA–H}]^-$ (*meta*-phenoxide isomer) at the RI-MP2/*aug*-cc-pVDZ level.

$E_{\text{RI-MP2}} = -646.4889858594$			
C	0.338504	−0.167727	0.000000
C	−0.929279	−0.817692	0.000000
C	−1.031946	−2.236426	0.000000
C	0.230544	−2.964050	0.000000
C	1.484106	−2.342216	0.000000
C	1.538659	−0.933950	0.000000
C	0.479196	1.274068	0.000000
C	−0.516787	2.217787	0.000000
C	−0.185985	3.638523	0.000000
O	0.057432	−4.315888	0.000000
O	−1.329673	4.427879	0.000000
O	0.926744	4.168338	0.000000
O	−2.103613	−2.967587	0.000000
H	−1.856234	−0.233754	0.000000
H	2.394712	−2.950888	0.000000
H	2.503255	−0.413861	0.000000
H	1.508137	1.661238	0.000000
H	−1.575270	1.950975	0.000000
H	−0.984502	5.337010	0.000000
H	−0.948000	−4.331780	0.000000

Table S6. Optimized Cartesian coordinates in Å and ground-state RI-MP2 energies, $E_{\text{RI-MP2}}$, in atomic units (a.u.) for the minimum-energy geometry of [FA–H]$^-$ (carboxylate C_1 isomer) at the RI-MP2/*aug*-cc-pVDZ level.

$E_{\text{RI-MP2}} = -685.6215463901$			
C	0.666084	0.165106	0.065664
C	−0.476247	−0.639661	0.307823
C	−0.379962	−2.029823	0.448559
C	0.875208	−2.670861	0.344776
C	2.019669	−1.893948	0.102453
C	1.913645	−0.497560	−0.033089
C	0.607754	1.628251	−0.090533
C	−0.502048	2.411352	−0.040776
C	−0.453975	3.933729	−0.227282
O	0.912232	−4.052035	0.497548
O	−1.592296	4.499260	−0.160417
O	0.690804	4.445005	−0.424796
H	−1.467539	−0.186903	0.402098
H	2.998556	−2.383803	0.025368
H	2.814642	0.095859	−0.219158
H	1.555639	2.151821	−0.271762
H	−1.497896	1.982814	0.134062
H	1.840878	−4.319353	0.441374
O	−1.520381	−2.777260	0.724823
C	−1.985848	−3.464282	−0.449848
H	−1.213189	−4.150538	−0.833167
H	−2.873141	−4.035655	−0.143238
H	−2.260827	−2.736588	−1.232790

Table S7. Optimized Cartesian coordinates in Å and ground-state RI-MP2 energies, $E_{\text{RI-MP2}}$, in atomic units (a.u.) for the minimum-energy geometry of $[\text{FA–H}]^-$ (carboxylate C_s isomer) at the RI-MP2/*aug*-cc-pVDZ level.

$E_{\text{RI-MP2}} = -685.6352859967$			
C	0.078443	1.045087	0.000000
C	0.024638	−0.374649	0.000000
C	1.203203	−1.129338	0.000000
C	2.469761	−0.499534	0.000000
C	2.539425	0.897236	0.000000
C	1.353099	1.657579	0.000000
C	−1.136802	1.878205	0.000000
C	−2.423599	1.440203	0.000000
C	−3.633154	2.382851	0.000000
O	3.625456	−1.260448	0.000000
O	−4.755914	1.781433	0.000000
O	−3.384768	3.627041	0.000000
H	−0.950510	−0.864579	0.000000
H	3.523668	1.374873	0.000000
H	1.412209	2.750581	0.000000
H	−0.998823	2.967329	0.000000
H	−2.663017	0.368155	0.000000
H	3.332334	−2.186942	0.000000
O	1.271602	−2.518520	0.000000
C	0.010008	−3.194734	0.000000
H	−0.571954	−2.937774	−0.900512
H	0.246651	−4.266281	0.000000
H	−0.571954	−2.937774	0.900512

Table S8. Optimized Cartesian coordinates in Å and ground-state RI-MP2 energies, $E_{\text{RI-MP2}}$, in atomic units (a.u.) for the minimum-energy geometry of $[\text{FA–H}]^-$ (phenoxide C_1 isomer) at the RI-MP2/*aug*-cc-pVDZ level.

$E_{\text{RI-MP2}} = -685.6444929588$			
C	0.659730	0.153162	−0.073351
C	−0.510236	−0.642404	−0.262359
C	−0.436481	−2.025352	−0.387828
C	0.831863	−2.763881	−0.343414
C	1.989279	−1.918591	−0.116200
C	1.907792	−0.533440	0.001451
C	0.632616	1.582380	0.050255
C	−0.458599	2.424695	0.002239
C	−0.288702	3.857609	0.151527
O	0.901886	−4.026697	−0.498130
O	−1.507570	4.532656	0.074024
O	0.750955	4.501514	0.328454
H	−1.501990	−0.180637	−0.326374
H	2.960021	−2.426058	−0.069828
H	2.820883	0.057078	0.153142
H	1.605477	2.071459	0.204053
H	−1.476087	2.057460	−0.147235
O	−1.621652	−2.713869	−0.634789
C	−1.911561	−3.669412	0.401140
H	−2.097052	−3.142452	1.355599
H	−2.827107	−4.192503	0.087174
H	−1.079164	−4.380151	0.509826
H	−1.256463	5.465079	0.186622

Table S9. Optimized Cartesian coordinates in Å and ground-state RI-MP2 energies, $E_{\text{RI-MP2}}$, in atomic units (a.u.) for the minimum-energy geometry of [FA–H]$^{-}$ (phenoxide C_s isomer) at the RI-MP2/*aug*-cc-pVDZ level.

$E_{\text{RI-MP2}} = -685.6456537231$			
C	0.940841	0.053854	0.000000
C	−0.293284	−0.674360	0.000000
C	−0.303185	−2.067856	0.000000
C	0.928875	−2.876424	0.000000
C	2.147101	−2.089391	0.000000
C	2.151480	−0.697160	0.000000
C	0.998254	1.482868	0.000000
C	−0.050736	2.384284	0.000000
C	0.202464	3.809068	0.000000
O	0.903606	−4.143904	0.000000
O	−0.985306	4.547093	0.000000
O	1.284447	4.408578	0.000000
H	−1.231121	−0.113507	0.000000
H	3.087065	−2.653168	0.000000
H	3.103915	−0.150987	0.000000
H	2.005441	1.924377	0.000000
H	−1.095909	2.067640	0.000000
O	−1.455297	−2.827944	0.000000
C	−2.671955	−2.093813	0.000000
H	−2.758947	−1.456483	−0.899631
H	−3.474113	−2.844503	0.000000
H	−2.758947	−1.456483	0.899631
H	−0.674688	5.468218	0.000000

S3. Photodepletion laser power dependence measurements

Laser power measurements were conducted on $[CMA–H]^–$, $[CA–H]^–$, and $[FA–H]^–$ at several of their respective absorption maxima when electrosprayed in either EtOH or MeCN. The plots displayed in Figures S1-S6 show the parent ion photodepletion intensities ($Int_{OFF} – Int_{ON}$) at the selected photon energies. Following standard protocols, the data has been plotted and fit to a power function.[1–3] The resultant slope is thereby proportional to the number of absorbed photons implicated in the experiment(s). Multiphoton events *via* instantaneous absorption of multiple photons in the Franck-Condon region are negligible as the laser beam is only softly focused through the ion-trap region. The slopes of all photon energies are less than 1.0, confirming that photodepletion of $[CMA–H]^–$, $[CA–H]^–$, and $[FA–H]^–$ at 0.3 mJ in both EtOH and MeCN are evidently not multiphoton in nature.

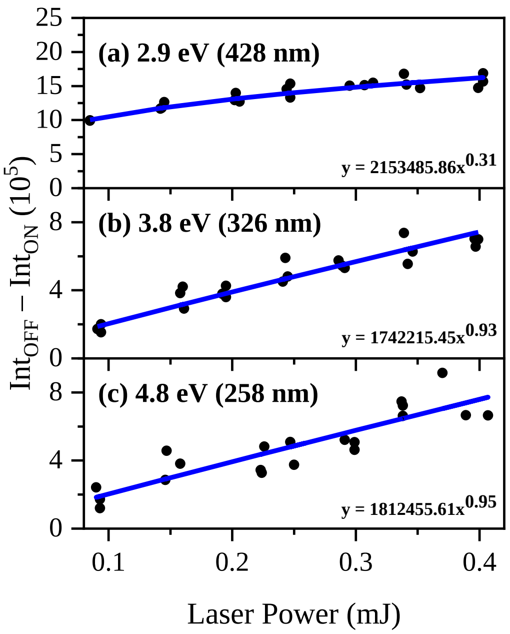

Figure S1. Power dependence measurements for $[CMA–H]^–$ at three absorption maxima of **(a)** 2.9 eV (428 nm), **(b)** 3.8 eV (326 nm), and **(c)** 4.8 eV (258 nm) when electrosprayed in EtOH.

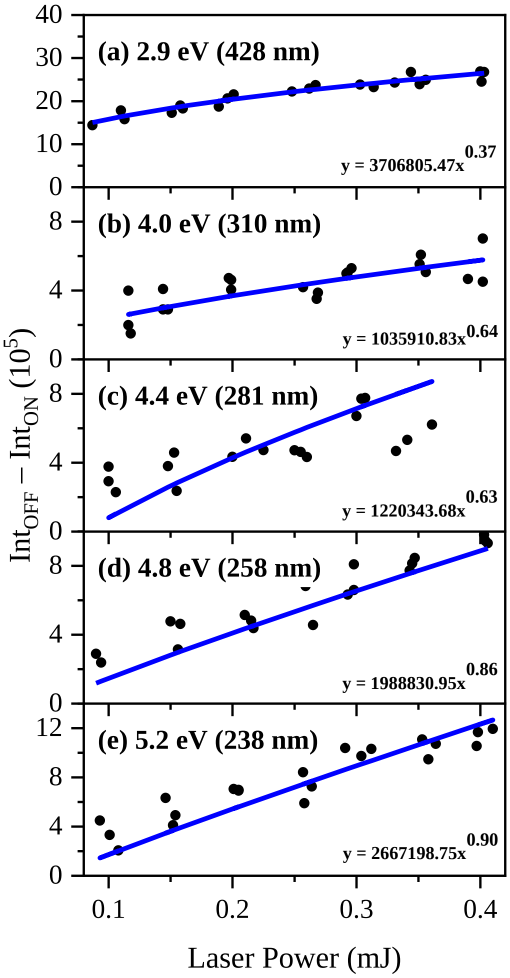

Figure S2. Power dependence measurements for [CA–H]⁻ at five absorption maxima of **(a)** 2.9 eV (428 nm), **(b)** 4.0 eV (310 nm), **(c)** 4.4 eV (281 nm), **(d)** 4.8 eV (258 nm), and **(e)** 5.2 eV (238 nm) when electrosprayed in EtOH.

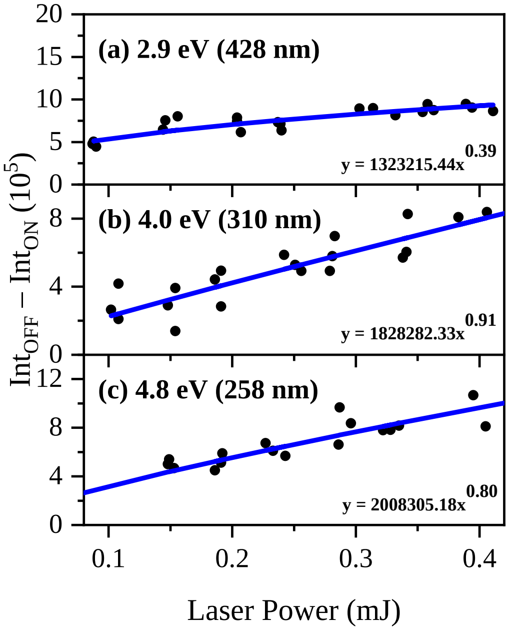

Figure S3. Power dependence measurements for $[FA–H]^{-}$ at three absorption maxima of **(a)** 2.9 eV (428 nm), **(b)** 4.0 eV (310 nm), and **(c)** 4.8 eV (258 nm) when electrosprayed in EtOH.

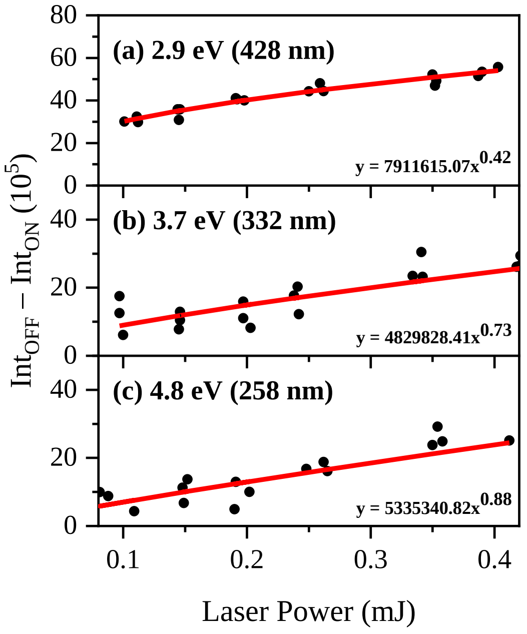

Figure S4. Power dependence measurements for [CMA–H]⁻ at three absorption maxima of **(a)** 2.9 eV (428 nm), **(b)** 3.7 eV (332 nm), and **(c)** 4.8 eV (258 nm) when electrosprayed in MeCN.

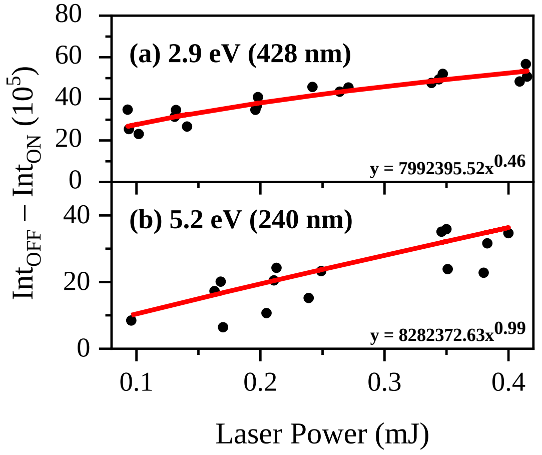

Figure S5. Power dependence measurements for [CA–H]$^-$ at three absorption maxima of **(a)** 2.9 eV (428 nm) and **(b)** 5.2 eV (240 nm) when electrosprayed in MeCN.

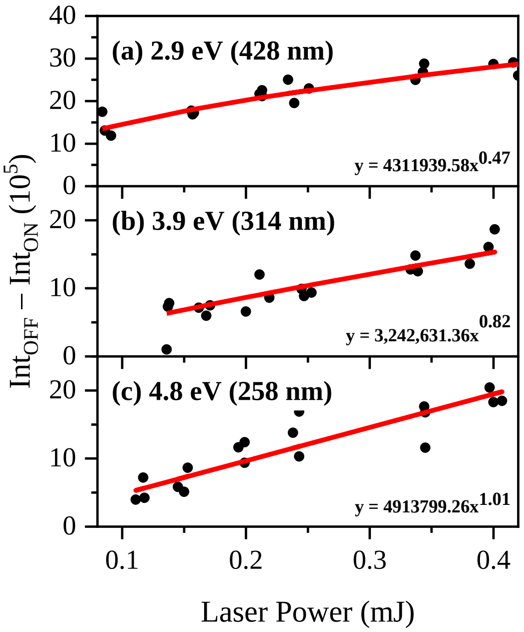

Figure S6. Power dependence measurements for [FA–H]$^{-}$ at three absorption maxima of **(a)** 2.9 eV (428 nm), **(b)** 3.9 eV (314 nm), and **(c)** 4.8 eV (258 nm) when electrosprayed in MeCN.

S4. Additional photofragment action spectra

Whilst the gas-phase UV-visible photofragmentation of $[CMA–H]^{-}$, $[CA–H]^{-}$, and $[FA–H]^{-}$ when electrosprayed in EtOH and MeCN proved not that all extensive, the photofragment action spectra of their respective minor fragments are displayed here in Section S4 to allow for a comprehensive assessment to be made across the photon energies scanned (2.5-5.5 eV). Notably, no minor photofragments were found for $[FA–H]^{-}$ when electrosprayed in MeCN.

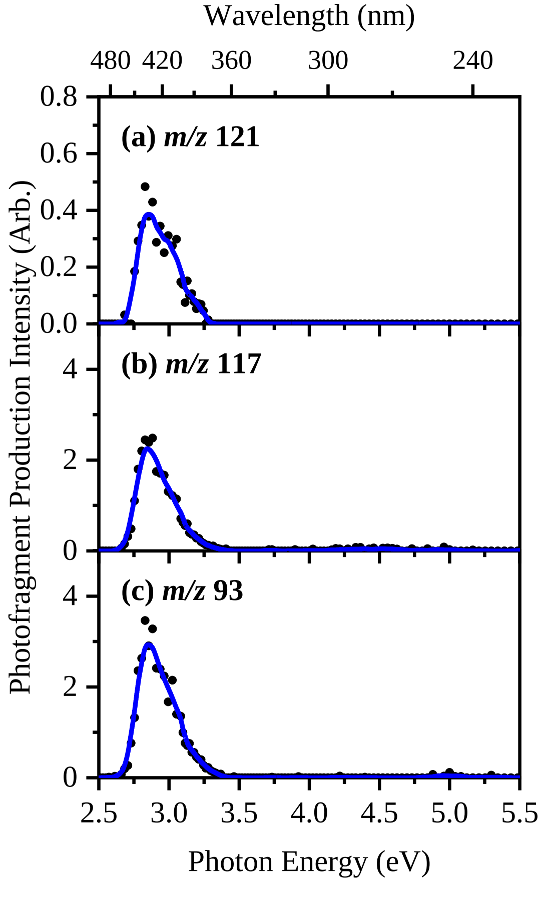

Figure S7. Additional photofragment action spectra for the minor fragments of $[CMA–H]^{-}$ observed at *m/z* 121, 117, and 93 when electrosprayed in EtOH. The solid line is a five-point adjacent average of the data points.

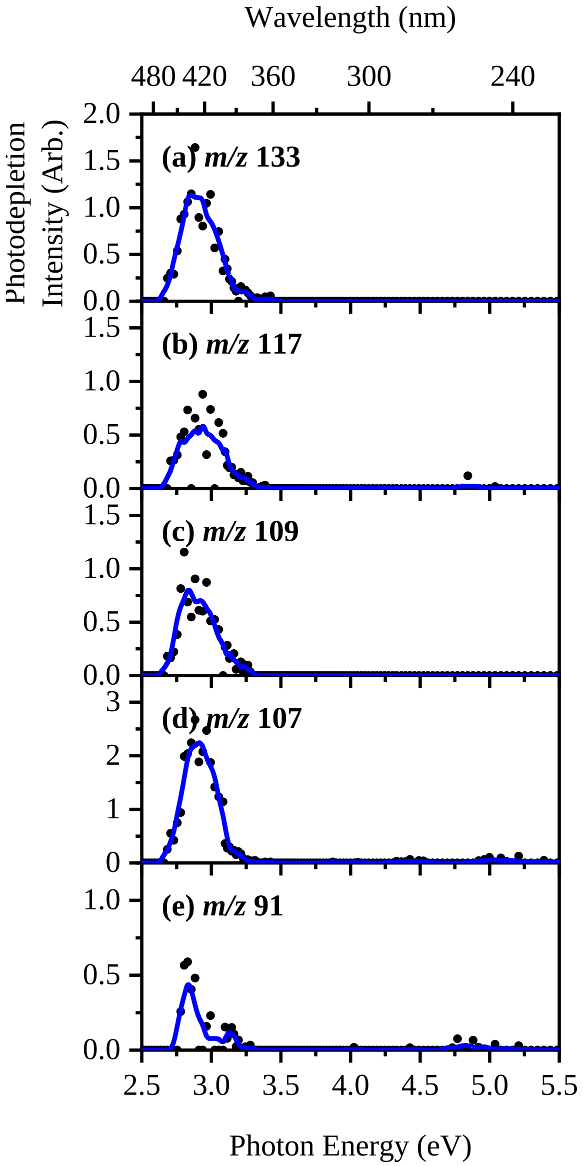

Figure S8. Additional photofragment action spectra for the minor fragments of $[CA{-}H]^-$ observed at *m/z* 133, 117, 109, 107, and 91 when electrosprayed in EtOH. The solid line is a five-point adjacent average of the data points.

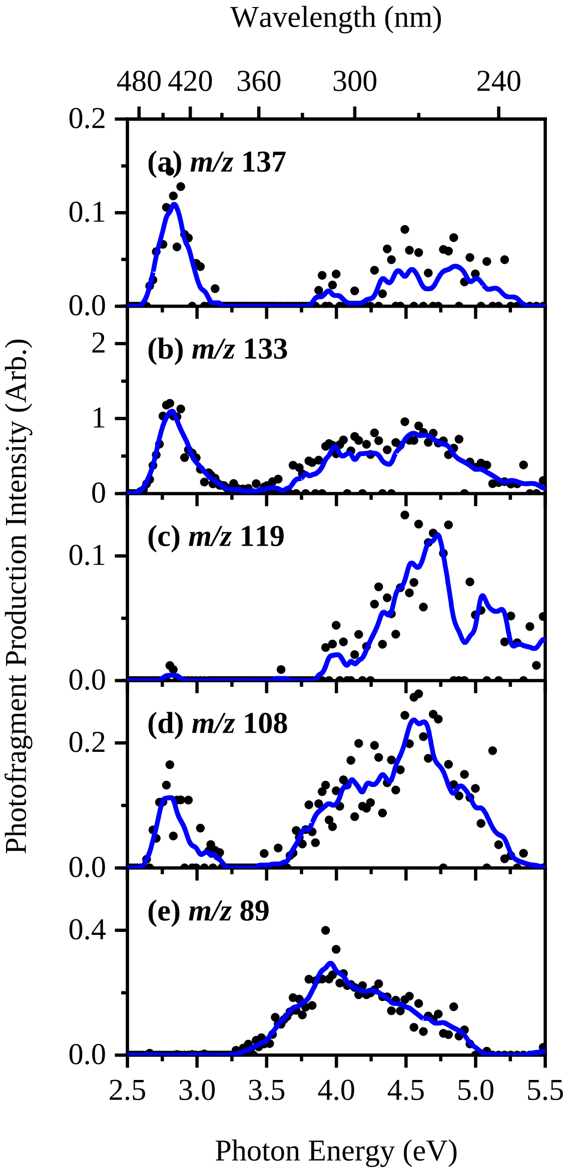

Figure S9. Additional photofragment action spectra for the minor fragments of [FA–H]$^-$ observed at *m/z* 137, 133, 119, 108, and 89 when electrosprayed in EtOH. The solid line is a five-point adjacent average of the data points.

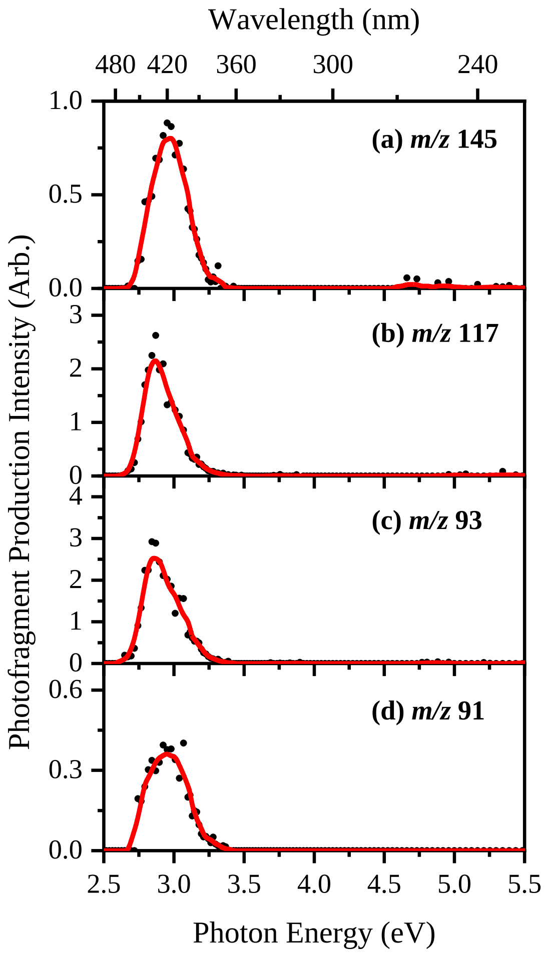

Figure S10. Additional photofragment action spectra for the minor fragments of $[CMA–H]^-$ observed at *m/z* 145, 117, 93, and 91 when electrosprayed in MeCN. The solid line is a five-point adjacent average of the data points.

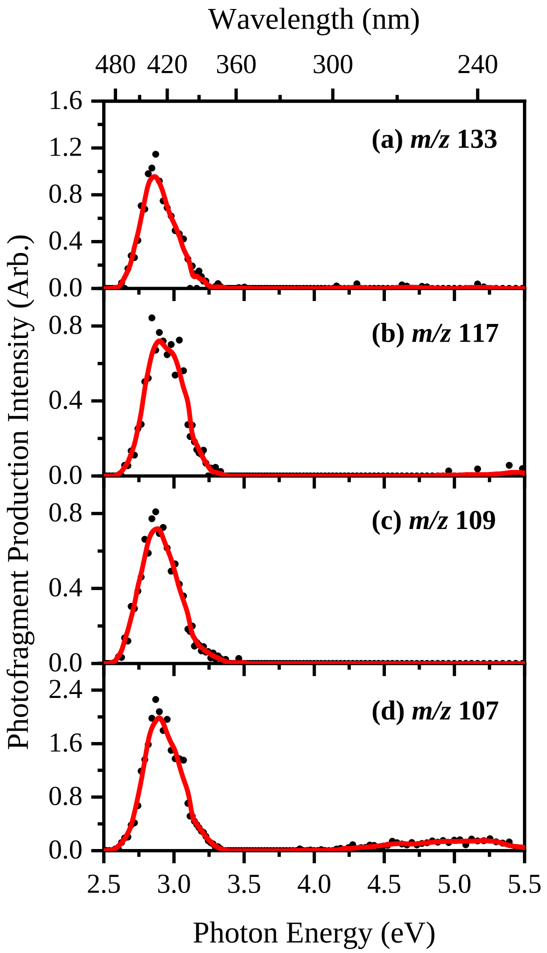

Figure S11. Additional photofragment action spectra for the minor fragments of [CA–H]⁻ observed at *m/z* 133, 117, 109, and 107 when electrosprayed in MeCN. The solid line is a five-point adjacent average of the data points.

S5. Ion yield plot

Figure S12 presents the relative photofragment ion yields of [FA–H]⁻ as a function of photoexcitation energy, highlighting several maxima that can be attributed to photoexcitation into different electronic states. It is evident that, the propensity of the relative ion yields of the photofragments at *m/z* 178, 149, 134, and 117 differ between electrospray of [FA–H]⁻ in EtOH and MeCN.

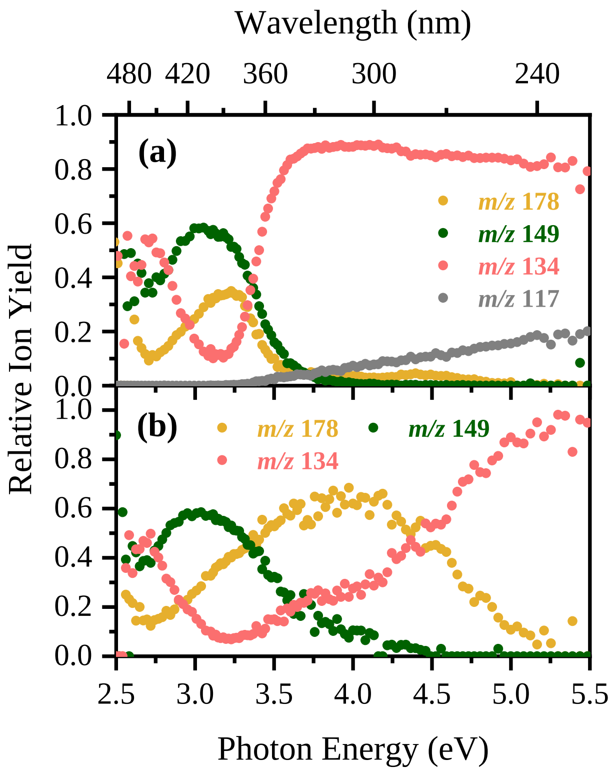

Figure S12. Relative ion yield plot highlighting the three most intense photofragments of [FA–H]⁻ at *m/z* 178, 149, 134, and 117 seen upon laser excitation between 2.5-5.5 eV when electrosprayed in **(a)** EtOH and **(b)** MeCN. It is of note that *m/z* 117 is only observed in solutions of [FA–H]⁻ electrosprayed in EtOH.

In EtOH (Figure S12a), *m/z* 149 dominates in production (*ca.* 60%) between 2.8-3.3 eV, with *m/z* 178 peaking at *ca.* 3.2 eV (36%). The ion at *m/z* 134 is observed to dominate in production beyond *ca.* 3.37 eV, where the production onset of the *m/z* 117 ion originates.

In MeCN, the *m/z* 149 ion dominates between 2.75-3.3 eV, before the *m/z* 178 ion dominates the ion yield between 3.3-4.3 eV, as displayed in Figure S12b. The ion at *m/z* 134 has a production onset of *ca.* 3.25 eV in the UV, peaking far beyond 5.5 eV (100%). Notably, the production of fragment ions in the UV are relatively low in MeCN in comparison to that of EtOH, which forms the *m/z* 134 fragment ion at high yields above 3.4 eV (see also Figure 4).

S6. Proposed Structures of Major Ionic Fragments

Table S10. Summary of the proposed structures of the major ionic fragments of electrosprayed [CMA–H]⁻, [CA–H]⁻, and [FA–H]⁻ produced upon HCD and UV-visible laser photoexcitation.

	**Ionic fragment *m/z* [a]**	**Proposed fragment(s)**	**Accompanying neutral fragment**
**[CMA–H]⁻ (*m/z* 163)**	119	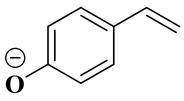	**CO_2**
**[CA–H]⁻ (*m/z* 179)**	135	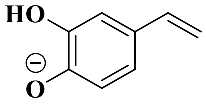 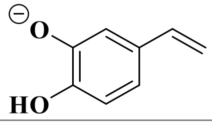	**CO_2**
	134	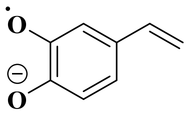 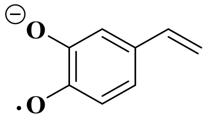	**CO_2 + H •**

Table S10. *Cont.*

	**Ionic fragment** *m/z* [a]	**Proposed fragment(s)**	**Accompanying neutral fragment**
**$[FA–H]^−$** **(*m/z* 193)**	178	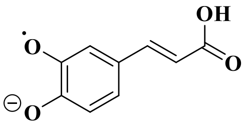 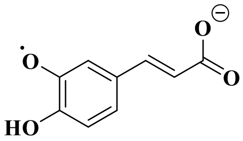	**CH_3 •**
	149	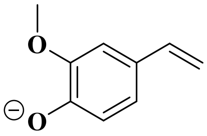	**CO_2**
	134	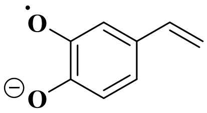	**CH_3 • + CO_2**

[a] Determined with mass accuracy > 0.3 amu.

S6. Additional calculation details: Excited state assignments

Table S11. Summary of states, characters,[a] symmetries, excitation energies (ΔE) in eV, and oscillator strengths (f)[b] for vertical transitions in $[CMA–H]^-$ at the minimum-energy geometries of the carboxylate (**1a**) and phenoxide (**1b**) deprotomers (Section S1; Scheme 1). ADC(2)/*aug*-cc-pVDZ.

Geom.	State	Char.	%	Sym.	ΔE / eV	f
**1a**	$^1n\pi^*$	$A''_6 \rightarrow A''_8$	48.2	A'	3.75	0.0172
		$A''_6 \rightarrow A''_9$	30.2			
	$^1\pi\pi^*$	$A''_7 \rightarrow A''_9$	44.8	A'	4.15	0.0427
		$A''_7 \rightarrow A''_8$	31.4			
	$^1n\pi^*$	$A''_6 \rightarrow A''_9$	72.0	A'	4.63	0.0370
	$^1\pi\pi^*$	$A''_7 \rightarrow A''_8$	22.9	A'	4.70	0.3446
		$A''_7 \rightarrow A''_9$	12.5			
**1b**	$^1\pi\pi^*$	$A''_7 \rightarrow A''_8$	90.7	A'	2.87	0.9729
	$^1\pi\pi^*$	$A''_7 \rightarrow A''_{11}$	42.3	A'	3.90	0.0940
		$A''_7 \rightarrow A''_{12}$	19.8			
	$^1\pi\pi^*$	$A''_6 \rightarrow A''_8$	89.4	A'	5.08	0.0489
	$^1\pi\pi^*$	$A''_7 \rightarrow A''_{12}$	45.5	A'	5.43	0.0270
		$A''_7 \rightarrow A''_{11}$	27.4			
	$^1(n/\pi)\pi^*$	$A''_5 \rightarrow A''_8$	65.6	A'	5.47	0.0687

[a] Configurations that contribute <10% to the state character are omitted. [b] Vertical transitions with an oscillator strength <0.015 are omitted.

Table S12. Summary of states, characters,[a] symmetries, excitation energies (ΔE) in eV, and oscillator strengths (f)[b] for vertical transitions in [CA–H]$^{-}$ at the minimum-energy geometries of the carboxylate (**2a**) and phenoxide (**2b**/**c**) deprotomers (Section 1; Scheme 1). ADC(2)/*aug*-cc-pVDZ.

Geom.	State	Char.	%	Sym.	ΔE / eV	f
**2a**	$^1n\pi^*$	$A''_7 \rightarrow A''_9$	48.2	A′	3.69	0.0172
		$A''_7 \rightarrow A''_{10}$	23.4			
	$^1\pi\pi^*$	$A''_8 \rightarrow A''_9$	52.7	A′	4.18	0.1401
		$A''_8 \rightarrow A''_{10}$	12.8			
	$^1\pi\pi^*$	$A''_8 \rightarrow A''_{11}$	28.5	A′	4.81	0.3940
		$A''_8 \rightarrow A''_9$	13.0			
		$A''_6 \rightarrow A''_9$	11.7			
**2b**	$^1\pi\pi^*$	$A''_8 \rightarrow A''_9$	90.7	A′	2.79	0.8960
	$^1\pi\pi^*$	$A''_7 \rightarrow A''_9$	33.1	A′	4.06	0.0218
		$A''_8 \rightarrow A''_{12}$	29.3			
		$A''_8 \rightarrow A''_{13}$	11.5			
	$^1\pi\pi^*$	$A''_7 \rightarrow A''_9$	53.7	A′	4.42	0.0456
		$A''_8 \rightarrow A''_{12}$	18.9			
		$A''_8 \rightarrow A''_{13}$	10.6			
	$^1\pi\pi^*$	$A''_8 \rightarrow A''_{10}$	47.0	A′	4.62	0.0251
		$A''_8 \rightarrow A''_{11}$	31.4			
	$^1(n/\pi)\pi^*$	$A''_6 \rightarrow A''_9$	73.2	A′	5.31	0.1440
**2c**	$^1\pi\pi^*$	$A''_8 \rightarrow A''_9$	88.2	A′	2.40	0.1710
	$^1\pi\pi^*$	$A''_7 \rightarrow A''_9$	80.3	A′	3.63	0.4208
	$^1\pi\pi^*$	$A''_8 \rightarrow A''_{12}$	18.0	A′	4.22	0.0976
		$A''_8 \rightarrow A''_{11}$	10.3			

Table S13. *Cont.*

Geom.	State	Char.	%	Sym.	ΔE / eV	f
**2c**	$^{1}\pi\pi^{*}$	$A''_8 \rightarrow A''_{12}$	13.8	A′	4.28	0.1358
		$A''_8 \rightarrow A''_{11}$	11.1			
	$^{1}\pi\pi^{*}$	$A''_8 \rightarrow A''_{10}$	29.3	A′	4.48	0.2591
		$A''_8 \rightarrow A''_{11}$	28.3			
		$A''_8 \rightarrow A''_{12}$	10.0			
	$^{1}\pi\pi^{*}$	$A''_8 \rightarrow A''_{10}$	25.0	A′	5.10	0.0408
		$A''_8 \rightarrow A''_{13}$	16.7			
		$A''_8 \rightarrow A''_{12}$	11.6			
	$^{1}(n/\pi)\pi^{*}$	$A''_6 \rightarrow A''_9$	47.8	A′	5.41	0.0266

[a] Configurations that contribute <10% to the state character are omitted. [b] Vertical transitions with an oscillator strength <0.015 are omitted.

Table S14. Summary of states, characters,[a] symmetries, excitation energies (ΔE) in eV, and oscillator strengths (f)[b] for vertical transitions in $[FA–H]^{–}$ at the minimum-energy geometries of the carboxylate (**3a**/**c**) and phenoxide (**3b**/**d**) deprotomers (Section S1; Scheme S1). ADC(2)/*aug*-cc-pVDZ.

Geom.	State	Char.	%	Sym.	ΔE / eV	f
**3a**	$^1n\pi^*$	$A''_8 \rightarrow A''_{11}$	57.2	A′	3.73	0.0168
		$A''_8 \rightarrow A''_{14}$	20.7			
	$^1\pi\pi^*$	$A''_9 \rightarrow A''_{11}$	49.8	A′	4.17	0.1777
		$A''_9 \rightarrow A''_{12}$	17.1			
	$^1n\pi^*$	$A''_8 \rightarrow A''_{10}$	84.2	A′	4.64	0.0246
	$^1\pi\pi^*$	$A''_9 \rightarrow A''_{14}$	29.0	A′	4.75	0.3158
		$A''_9 \rightarrow A''_{11}$	15.3			
**3b**	$^1\pi\pi^*$	$A''_9 \rightarrow A''_{11}$	89.9	A′	2.76	0.9538
	$^1\pi\pi^*$	$A''_9 \rightarrow A''_{10}$	87.0	A′	3.70	0.0132
	$^1\pi\pi^*$	$A''_9 \rightarrow A''_{15}$	36.9	A′	4.08	0.0544
		$A''_9 \rightarrow A''_{14}$	12.1			
	$^1\pi\pi^*$	$A''_8 \rightarrow A''_{11}$	75.9	A′	4.56	0.0205
	$^1(n/\pi)\pi^*$	$A''_7 \rightarrow A''_{11}$	60.1	A′	5.30	0.0706
**3c**	$^1n\pi^*$	$A_{50} \rightarrow A_{64}$	31.6	A	3.69	0.0161
	$^1\pi\pi^*$	$A_{51} \rightarrow A_{64}$	27.0	A	4.16	0.1047
	$^1\pi\pi^*$	$A_{51} \rightarrow A_{65}$	16.6	A	4.84	0.2213
	$^1n\pi^*$	$A_{50} \rightarrow A_{65}$	23.1	A	4.86	0.0601

Table S14. *Cont.*

Geom.	State	Char.	%	Sym.	**Δ*E* / eV**	***f***
	$^1\pi\pi^*$	$A_{51} \rightarrow A_{61}$	87.9	A	2.79	0.9319
**3d**	$^1\pi\pi^*$	$A_{50} \rightarrow A_{61}$	72.0	A	4.61	0.0241
	$^1(n/\pi)\pi^*$	$A_{48} \rightarrow A_{61}$	70.8	A	5.30	0.1057

[a] Configurations that contribute <10% to the state character are omitted. [b] Vertical transitions with an oscillator strength <0.015 are omitted.

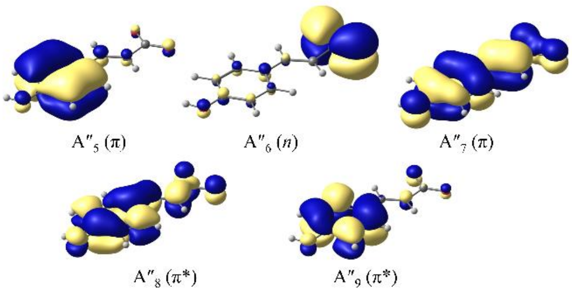

Figure S13. Illustration of the key molecular orbitals; carboxylate deprotomer of [CMA–H]$^-$ (**1a**: Section S1; Scheme S1). MP2/*aug*-cc-pVDZ.

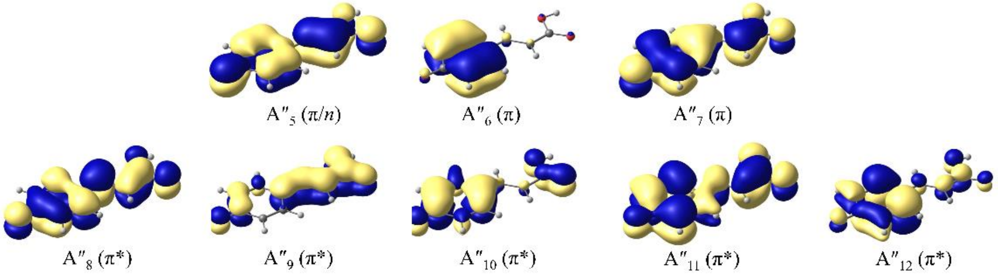

Figure S14. Illustration of the key molecular orbitals; phenoxide deprotomer of [CMA–H]$^-$ (**1b**: Section S1; Scheme S1). MP2/*aug*-cc-pVDZ.

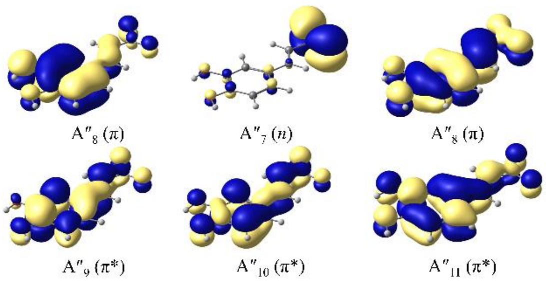

Figure S15. Illustration of the key molecular orbitals; carboxylate deprotomer of [CA–H]$^-$ (**2a**: Section S1; Scheme S1). MP2/*aug*-cc-pVDZ.

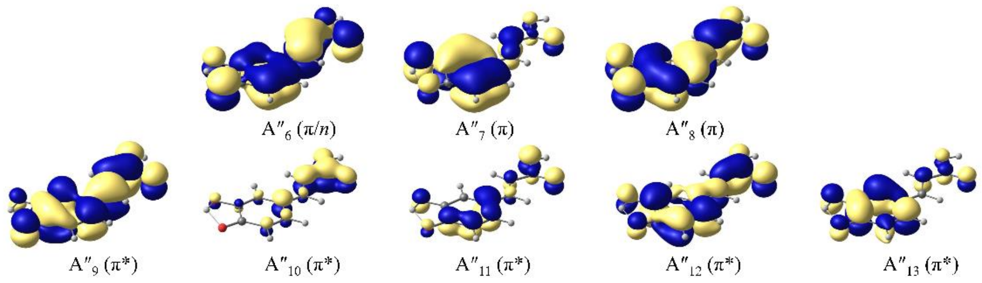

Figure S16. Illustration of the key molecular orbitals; *para*-phenoxide deprotomer of [CA–H]$^-$ (**2b**: Section S1; Scheme S1). MP2/*aug*-cc-pVDZ.

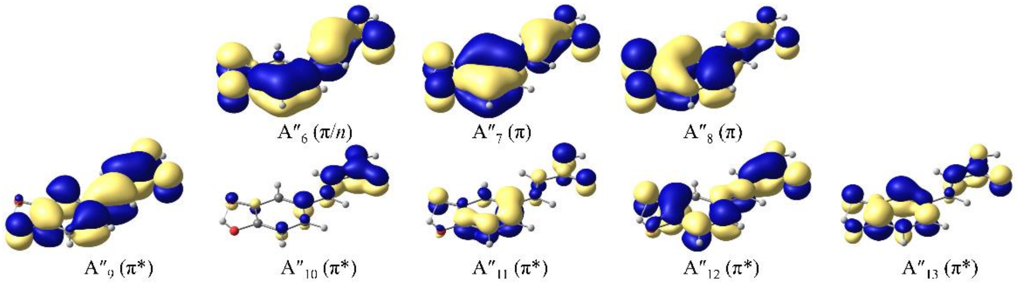

Figure S17. Illustration of the key molecular orbitals; *meta*-phenoxide deprotomer of [CA–H]$^-$ (**2c**: Section S1; Scheme S1). MP2/*aug*-cc-pVDZ.

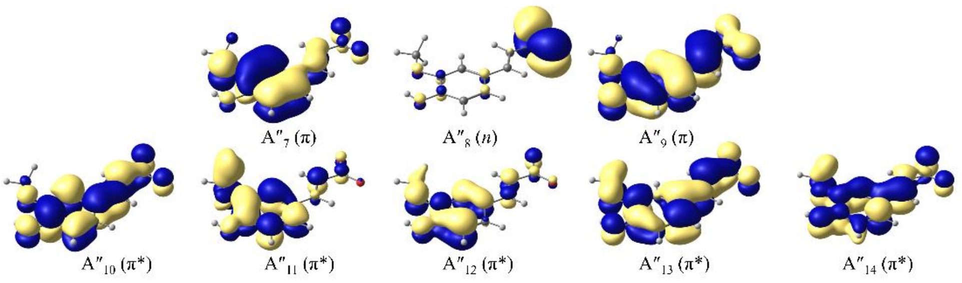

Figure S18. Illustration of the key molecular orbitals; C_s-symmetric carboxylate deprotomer of [FA–H]$^-$ (**3a**: Section Se1; Scheme S1). MP2/*aug*-cc-pVDZ.

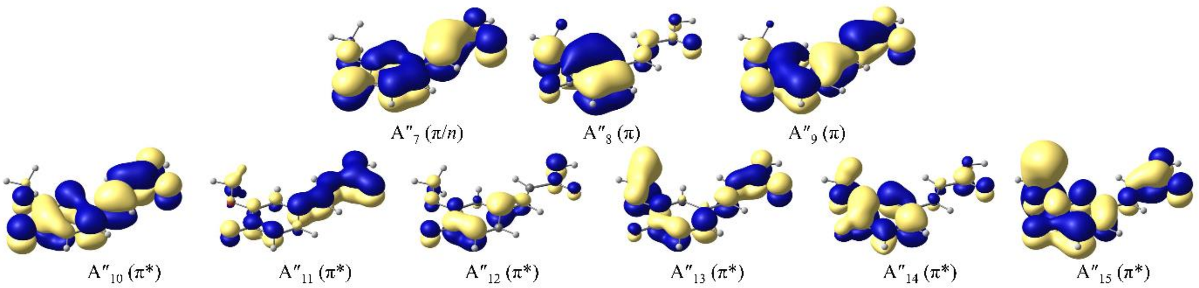

Figure S19. Illustration of the key molecular orbitals; C_s-symmetric phenoxide deprotomer of $[FA–H]^-$ (**3b**: Section S1; Scheme S1). MP2/*aug*-cc-pVDZ.

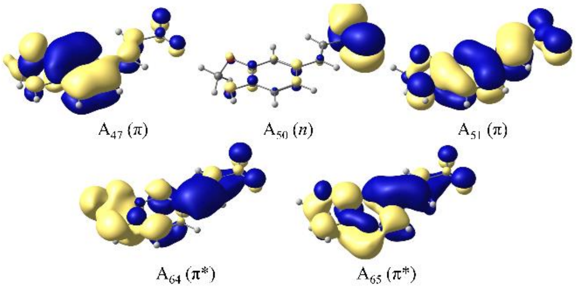

Figure S20. Illustration of the key molecular orbitals; C_1-symmetric carboxylate deprotomer of $[FA–H]^-$ (**3c**: Section S1; Scheme S1). MP2/*aug*-cc-pVDZ.

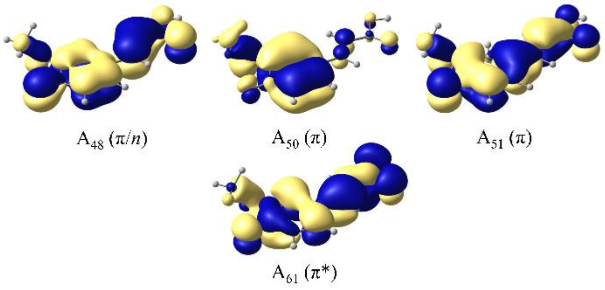

Figure S21. Illustration of the key molecular orbitals; C_1-symmetric phenoxide deprotomer of $[FA–H]^-$ (**3d**: Section S1; Scheme S1). MP2/*aug*-cc-pVDZ.

References

(1) Wong, N. G. K.; Rankine, C. D.; Dessent, C. E. H. Linking Electronic Relaxation Dynamics and Ionic Photofragmentation Patterns for the Deprotonated UV Filter Benzophenone-4. *J. Phys. Chem. Lett.* **2021**, *12*, 2831–2836.

(2) Berenbeim, J. A.; Wong, N. G. K.; Cockett, M. C. R.; Berden, G.; Oomens, J.; Rijs, A. M.; Dessent, C. E. H. Unravelling the Keto–Enol Tautomer Dependent Photochemistry and Degradation Pathways of the Protonated UVA Filter Avobenzone. *J. Phys. Chem. A* **2020**, *124*, 2919–2930.

(3) Wong, N. G. K.; Berenbeim, J. A.; Dessent, C. E. H. Direct Observation of Photochemical Free Radical Production from the Sunscreen 2-Phenylbenzimidazole-5-Sulfonic Acid via Laser-Interfaced Mass Spectrometry. *ChemPhotoChem* **2019**, *3*, 1231–1237.
